# Administration of the K_Ca_ channel activator SKA-31 improves endothelial function in the aorta of atherosclerosis-prone mice

**DOI:** 10.3389/fphar.2025.1545050

**Published:** 2025-02-28

**Authors:** O. Daniel Vera, Ramesh C. Mishra, Rayan Khaddaj-Mallat, Liam Hamm, Barak Almarzouq, Yong-Xiang Chen, Darrell D. Belke, Latika Singh, Heike Wulff, Andrew P. Braun

**Affiliations:** ^1^ Department of Physiology and Pharmacology, Cumming School of Medicine, University of Calgary, Calgary, AB, Canada; ^2^ Libin Cardiovascular Institute, Cumming School of Medicine, University of Calgary, Calgary, AB, Canada; ^3^ Department of Pharmacology, School of Medicine, University of California Davis, Davis, CA, United States

**Keywords:** endothelial dysfunction, KCa channel activator, aorta, atherosclerosis, ApoE knockout mouse

## Abstract

**Introduction:**

Atherosclerosis remains a major risk factor for vascular dysfunction and cardiovascular (CV) disease. Pharmacological enhancement of endothelial Ca^2+^-activated K^+^ channel activity (i.e., K_Ca_2.3 and K_Ca_3.1) opposes vascular dysfunction associated with ageing and type 2 diabetes (T2D) in *ex vivo* and *in vivo* preparations. In the current study, we have investigated the efficacy of this strategy to mitigate endothelial dysfunction in the setting of atherogenesis.

**Methods:**

Male apolipoprotein E knockout (Apoe^−/−^) mice fed a high fat diet (HFD) were treated daily with the K_Ca_ channel activator SKA-31 (10 mg/kg), the K_Ca_3.1 channel blocker senicapoc (40 mg/kg), or drug vehicle for 12-weeks. Endothelium-dependent and -independent relaxation and vasocontractility were measured in abdominal aorta by wire myography. The development of atherosclerosis in the thoracic aorta was characterized by Oil Red O staining and immunohistochemistry. Key vasorelaxant signaling proteins were quantified by q-PCR.

**Results:**

Endothelium-dependent relaxation of phenylephrine-constricted aortic rings was impaired in Apoe^−/−^ HFD mice (53%) vs. wild-type (WT) controls (80%, P < 0.0001), consistent with endothelial dysfunction. Treatment of Apoe^−/−^ HFD mice with SKA-31, but not senicapoc, restored maximal relaxation to the WT level. Phenylephrine-evoked contraction was similar in WT and vehicle/drug treated Apoe^−/−^ mice, as was the maximal relaxation induced by the endothelium-independent vasodilator sodium nitroprusside. mRNA expression for eNOS, K_Ca_3.1, K_Ca_2.3 and TRPV4 channels in the abdominal aorta was unaffected by either SKA-31 or senicapoc treatment. Fatty plaque formation, tissue collagen, α-smooth muscle actin and resident macrophages in the aortic sinus were also unaltered by either treatment vs. vehicle treated Apoe^−/−^ HFD mice.

**Conclusion:**

Our data show that prolonged administration of the K_Ca_ channel activator SKA-31 improved endothelial function without modifying fatty plaque formation in the aorta of Apoe^−/−^ mice.

## 1 Introduction

Atherosclerosis is a vascular disease induced by high levels of low-density lipoprotein cholesterol (LDL-C) circulating in the blood (Libby et al., 2019). After LDL-C undergoes oxidative modifications by reactive oxygen species in the vascular wall, it can induce morphological changes to the endothelium, including increased endothelial expression of cell adhesion molecules and secretion of pro-inflammatory cytokines (Libby et al., 2019). These morphological changes to endothelial cells contribute to endothelial dysfunction (Hadi et al., 2005) that further promotes leukocyte recruitment, vascular inflammation, disruption of vasodilatory signaling, and atherosclerotic fatty plaque formation (Hadi et al., 2005). Subsequently, atherosclerotic fatty plaques may become unstable, burst and release factors into the circulation to induce thrombosis and major acute cardiovascular events such as myocardial infarction and stroke (Libby, 2013; Campbell et al., 2019).

Ca^2+^-activated K^+^ (K_Ca_) channels are expressed in a variety of cell types, including endothelial cells and immune cells, where they contribute to the regulation of membrane potential and Ca^2+^ signaling (Feske et al., 2015; Köhler et al., 2016). Notably, endothelial K_Ca_2.3 and K_Ca_3.1 channel activity contributes to endothelial-derived hyperpolarization (EDH) and vasodilatory mechanisms that promote healthy endothelial function (Köhler et al., 2016; Vanhoutte et al., 2017). Previous studies from our group have shown that administration of the K_Ca_2.3/K_Ca_3.1 channel activator SKA-31 (Sankaranarayanan et al., 2009) can restore endothelial function in arteries from aged (Mathew John et al., 2020) and type 2 diabetic rats (Mishra et al., 2021; Mishra et al., 2024) that exhibit endothelial dysfunction. However, it remains unknown whether enhancement of K_Ca_2.3/K_Ca_3.1 channel activity *in vivo* can improve endothelial function under conditions of atherogenesis. Previous *in vivo* mouse studies have shown that the K_Ca_3.1 channel blocker TRAM-34 can decrease aortic atherosclerosis development, in part by inhibiting pro-inflammatory cytokine secretion by macrophages and de-differentiation and proliferation of vascular smooth muscle cells (VSMCs) (Toyama et al., 2008; Xu et al., 2017; Tharp and Bowles, 2021; Jiang et al., 2022). However, these studies using TRAM-34 did not examine potential changes in endothelial function and vascular reactivity, which are known to impact blood pressure regulation and cardiovascular health. Therefore, the objective of the present study was to investigate the potential beneficial effect of SKA-31 administration on endothelial function in male apolipoprotein E knockout (Apoe^−/−^) mice maintained on a high fat diet (HFD). Parallel treatment of Apoe^−/−^ HFD mice with senicapoc (ICA-17043), a potent and selective K_Ca_3.1 channel blocker (McNaughton-Smith et al., 2008) was carried out for comparison purposes.

## 2 Materials and Methods

### 2.1 Experimental animals and treatment outline

Atherosclerosis-prone male Apoe^−/−^ mice on a C57BL/6 genetic background (JAX strain B6.129P2-Apoe^tm1unc^/J; JAX stock # 002052) were purchased from the Jackson Laboratory (Bar Harbor, ME, United States). Beginning at 8 weeks of age, Apoe^−/−^ mice were fed the Envigo TD.88137 high fat diet (HFD) with 21.2% fat and 0.2% cholesterol by weight (Envigo, Indianapolis, IN, United States), and were maintained on this diet until they were euthanized. Male wild-type (WT) C57BL/6N mice were purchased from Charles River (Wilmington, MA, United States) at 20–22 weeks of age and served as a healthy reference group for Apoe^−/−^ HFD mice. WT mice were fed a regular chow diet (Pico-Vac Mouse Diet 20 Irradiated, PM Nutrition International, Arden Hills, MN, United States). All mice were housed at 22°C under a 12 h light/12 h dark cycle and had access to food and water *ad libitum*. Animal procedures were reviewed and approved by the University of Calgary Animal Care Committee (Protocol # AC19-0056). Four groups of animals (62 mice in total) were used in this project:

1) C57BL/6N (WT) + Normal Diet
2) Apoe^−/−^ with Vehicle (Miglyol 812N) + HFD
3) Apoe^−/−^ with SKA-31 (10 mg/kg/day) + HFD
4) Apoe^−/−^ with senicapoc (40 mg/kg/day) + HFD

Treatment with the K_Ca_3.1 channel blocker senicapoc was used as a benchmark for the *in vivo* actions of K_Ca_2.3/K_Ca_3.1 channel activator SKA-31, based on earlier reports that a K_Ca_3.1 inhibitor mitigates atherogenesis in Apoe^−/−^ mice (Toyama et al., 2008; Tharp and Bowles, 2021; Jiang et al., 2022). SKA-31 and senicapoc were synthesized and prepared as stock solutions in Miglyol 812N (OIO Oleochemical, Hamburg, Germany) as previously described (Sankaranarayanan et al., 2009; Jin et al., 2019; Mathew John et al., 2020). Aliquots of the dissolved compounds (50 µL each) were mixed with ∼150 µL of condensed milk to achieve the desired experimental dosage and delivered to mice in a plastic tray for ingestion; compounds were administered daily between 12p.m. and 4p.m. for 12 weeks. At ∼20 weeks of age, all Apoe^−/−^ mice were euthanized, following 12 weeks of treatment with vehicle, SKA-31, or senicapoc. WT mice were euthanized at 20–22 weeks of age. Following euthanasia, the total plasma concentration of senicapoc in treated Apoe^−/−^ mice was determined with the same method and instrumentation as previously described (Jin et al., 2019).

### 2.2 Analyses of serum lipids and cytokines

Following euthanasia with carbon dioxide and cardiac puncture, ∼300 µL of blood was put into a serum separator tube (SST). Following centrifugation at ∼1200xg at 4°C for 10 min, clear serum was collected and stored at −80°C until chemical analysis. Serum samples (∼70 µL) were analyzed by IDEXX Bio-Analytics (Sacramento, CA, United States) for total cholesterol (TC), high-density lipoprotein cholesterol (HDL-C), triglycerides, and low-density lipoprotein cholesterol (LDL-C) using an AU Beckman Coulter Analyzer. Another aliquot of serum was analyzed for select cytokines using a Luminex xMAP fluorescence-based, multi-plex assay (EVE Technologies, Calgary, Canada).

### 2.3 Isolation of the aorta and other organs

Following euthanasia, the abdominal aorta was removed and stored in ice-cold Krebs-Henseleit solution (in mM: 120 NaCl, 20 NaHCO_3_, 4.8 KCl, 1.2 NaH_2_PO_4_, 1.2 MgSO_4_, 11 Glucose, 2.5 CaCl_2_, adjusted to pH 7.4). Then, 4% paraformaldehyde (PFA) in 1x phosphate-buffered saline (PBS) was injected through the left ventricle of the heart to chemically fix the thoracic aorta, which was then separated from the heart and stored in 4% PFA. Additional organs including the heart, brain, kidneys, and liver were then harvested and fixed in 4% PFA prior to histological analyses. The dry weight of each heart was recorded. The left leg of each mouse was also collected; the tibia was isolated from the leg with 5 M NaOH and heating, and the tibial length was recorded.

### 2.4 Oil Red O staining of the thoracic aorta

The thoracic aorta was prepared by cutting it open to expose the luminal surface and then placed *en face* on a glass slide, covered with plastic wrap and allowed to rest overnight at room temperature (20°C). The thoracic aorta was then treated with 0.5% Oil Red O 1,2-propanediol solution (Sigma Aldrich) for 10 min at 65°C to stain atherosclerotic lesions, as described previously (Lin et al., 2015; Andrés-Manzano et al., 2105). Digital images of the *en face* thoracic aorta were collected using an Olympus SZ-61 stereo microscope equipped with an Olympus UC30 camera (Olympus Corp., Tokyo, Japan) at ×12 magnification. The severity of atherosclerotic lesions was measured in the descending thoracic aorta and the aortic arch where the most prominent fatty plaques are observed in Apoe^−/−^ mice (Nakashima et al., 1994). The total area of the thoracic aorta and observed lesion areas (identified by visible red staining) were manually measured using the polygon selection tool in ImageJ software (V1.53a, NIH, Bethesda, MD, United States), and data are presented as percent aortic area occupied by lesions. Care was taken to ensure that advanced lesions (i.e., a white region surrounded by red staining) were also included in the positive lesion area measurement.

### 2.5 Hematoxylin and eosin (H&E) histology of select organs

The heart, brain, kidneys, and liver tissues stored in 4% PFA were dehydrated, embedded in paraffin wax, and then cut into 5 µm-thick sections using a microtome. The slide-mounted sections were then stained with hematoxylin and eosin (H&E) to visualize general tissue morphology, as previously described (Seibert et al., 2013). The stained sections were visualized using appropriate magnification with an Olympus BX53 fluorescence-equipped microscope (Olympus Corp., Tokyo, Japan) under brightfield illumination. Morphological analyses were done with the use of ImageJ software.

### 2.6 Immunohistochemical detection of resident macrophages

Sections of the aortic sinus (5 µm thick) were analyzed for inflammatory macrophage content by staining with an antibody recognizing the Mac-2 antigen (also known as galectin-3) (Sundblad et al., 2011), as described (Chen et al., 2021). Following deparaffinization, aortic sinus sections were blocked with 10% normal horse serum (Thermo Fisher Scientific, Catalog # 31874) in 1 × PBS for 10 min at room temperature (RT, ∼21°C). Sections were then incubated at 4°C overnight with rat anti-mouse macrophage primary antibody (Mac-2, Accurate Chemical and Scientific Corp., Catalog # ACL8942AP, stock concentration 1 mg/mL) diluted 1:500 in 1x PBS. Sections were then incubated with biotinylated rabbit anti-rat IgG (H + L) secondary antibody (Vector Laboratories, Catalog # BA-4000-1.5, stock concentration 1.5 mg/mL) diluted 1:100 in 1 × PBS. Endogenous peroxidase activity was quenched with 3% H_2_O_2_ for 20 min at RT. Antibody reactivity was then detected with a Vectastain Elite ABC-HRP Kit (Vector Laboratories, Catalog # PK-6100), along with diaminobenzidine (DAB) as the peroxidase substrate (Sigma Aldrich, Catalog #D5905-50TAB). Finally, tissue sections were counterstained with hematoxylin and visualized at ×40 magnification under brightfield illumination. Mac-2-positive area (i.e., area stained with brown color) is reported as a percentage of the total aortic sinus area and was analyzed with the use of ImageJ software.

### 2.7 Measurement of vasoactive responses in the abdominal aorta by wire myography

Following isolation, the abdominal aorta was placed in cold Krebs-Henseleit buffer (in mM: 120 NaCl, 20 NaHCO_3_, 4.8 KCl, 1.2 NaH_2_PO_4_, 1.2 MgSO_4_, 11 Glucose, 2.5 CaCl_2_, adjusted to pH 7.4), and ∼2 mm ring segments were prepared from the aortic segment located between the renal arteries and the iliac bifurcation. Typically, two aortic rings could be prepared from each animal, which were mounted individually in bath chambers on parallel pins connected to a force transducer (Model 620 M Multi Wire Myograph System, Danish Myo Technology, Hinnerup, Denmark). The Krebs-Henseleit buffer solution in the bath chambers was maintained at 37°C and bubbled with 95% air/5% CO_2_ gas. The wire myography instrument was connected to a digital computer interface and data were collected on an Acer Aspire XC-866 Series computer using LabChart 8 software (AD Instruments Inc, Colorado Springs, CO, United States). Abdominal aortic rings were first equilibrated at 1 gm of tension for 45 min, during which the Krebs-Henseleit buffer was replaced every 10 min. Then, a buffer solution containing 60 mM KCl (in mM: 64.8 NaCl, 20 NaHCO_3_, 60 KCl, 1.2 NaH_2_PO_4_, 1.2 MgSO_4_, 11 Glucose, 2.5 CaCl_2_, adjusted to pH 7.4) was added to the bath to evaluate smooth muscle contractility. Following KCl washout and recovery of basal tension, a cumulative concentration-response experiment was carried out with increasing concentrations of phenylephrine (PE) (final concentrations: 0.1, 1, 10, 100, 1000 and 10,000 nM) added to the bath every 5 min. Following washout of PE and recovery of basal tension, vessels were pre-constricted with 1 μM PE to establish steady-state tone and then a cumulative concentration-response experiment was carried out with the endothelium-dependent vasodilatory agonist acetylcholine (ACh), which was added in increasing concentrations every 5 min (final concentrations: 1, 3, 10, 100, 1000 and 10,000 nM). After washout of both PE and ACh, aortic rings were again pre-constricted with 1 μM PE and a cumulative concentration-response experiment was carried out with the endothelium-independent vasodilator sodium nitroprusside (SNP) (0.1, 1, 3, 10, 100 and 1000 nM final concentrations). After washout of both PE and SNP, aortic constriction was induced again with 1 μM PE to confirm that the relaxation response induced by SNP was reversible and the tissues remained viable throughout the protocol.

For wire myography, pooled data for concentration-response curves (CRCs) for PE, ACh, and SNP were generated and plotted on semi-logarithmic graphs, where each animal is represented by one abdominal aortic ring. PE-induced contraction was normalized to the maximal contraction evoked by 60 mM KCl, whereas ACh and SNP-induced relaxations were normalized to the vessel’s pre-constriction level induced by 1 μM PE. Using GraphPad Prism Version 10 (Dotmatics, San Diego, CA, United States), the concentration-response data were used to calculate the maximal response (E_max_) and the potency (logEC_50_) of PE, ACh, and SNP by curve fitting the CRC for each individual vessel using the nonlinear regression log (agonist) vs. response (three parameters) method. The equation for the log (agonist) vs. response (three parameters) method is:

$$Y=\frac{Bottom+\left(Top-Bottom\right)}{1+10^{logEC50-X}}$$

where X is the logarithm of the agonist concentration and Y is the response. This equation provided a logEC_50_ and a top bound (E_max_) value for each CRC. These logEC_50_ and E_max_ values were then pooled to acquire mean values for logEC_50_ and E_max_ for all four groups.

### 2.8 Echocardiography, left ventricular function and aortic wall stiffness

Doppler echocardiography was carried out by using the Vevo 3100 Imaging System (VisualSonics, Toronto, ON, Canada). The echocardiography measurements were executed at baseline and terminal (post-treatment) points in a blinded manner by Dr. D Belke (Libin CV Institute physiology core facility). Mice were anesthetized with 4% isoflurane with oxygen as the carrier gas and followed by a decrease to 1.5%–2% to achieve light anesthesia. Each mouse was placed supine on a heated pad (∼37°C) and paws were taped to electrocardiogram (ECG) tracing panels following application of electrode gel (Signa Gel, Parker Laboratories, Fairfield, NJ, United States) on the ECG tracing panels, Hair removal gel (Nair, Church & Dwight, Ewing, NJ, United States) was used to remove hair from the stomach and thoracic area of the animal and the gel and hair was wiped off after ∼1 min of application. The area was then cleaned with 75% ethanol, and Aquasonic 100 Ultrasound Transmission Gel (Parker Laboratories, Fairfield, NJ, United States) was placed on the animal’s stomach to facilitate use of the measuring probe.

One-dimensional M-mode echocardiography was used to measure heart rate (HR), left ventricle (LV) wall thickness, ejection fraction (EF), and fractional shortening (FS), followed by two-dimensional B-mode echocardiography to obtain a longitudinal view of the LV. ECG-gated kilohertz visualization was used to create a 3D reconstruction of the heart to allow for LV volume measurements including stroke volume (SV), end-diastolic volume (EDV), end-systolic volume (ESV), and cardiac output (CO). The pulse wave (PW) Doppler mode was then used to measure pulse waves at the aortic root and the femoral artery; pulse wave velocity was then calculated by using a transit-time approach similar to protocols described previously (Hartley et al., 2000; Williams et al., 2007), based on the following equation:

$$PWV=\frac{d_{2}-d_{1}}{t_{2}-t_{1}}$$

where d_2_ – d_1_ represents the distance between the aortic root and the femoral artery, and t_2_ – t_1_ represents the difference between the pulse wave times at the femoral artery and the aortic root. The distances between the aortic root to the aortic bifurcation and the aortic bifurcation to femoral artery were measured through the study by the same blinded colleague to maintain consistency of measurements.

### 2.9 RNA extraction and quantitative PCR

Total RNA was isolated from flash frozen abdominal aortic rings using the RNeasy Micro Kit (Qiagen cat # 74004), according to the manufacturer’s instructions. Total RNA from lung and kidney tissue was extracted to act as a positive control; for negative control samples, buffer was added in place of a complementary DNA (cDNA) template. The concentration and integrity of extracted total RNA were assessed using a Tape Station assay (University of Calgary Genomic Services). cDNA was synthesized from total RNA using a QuantiTect Reverse Transcription Kit (Qiagen cat # 205311). Quantitative RT-PCR was performed using the PowerUp™ SYBR™ Green Master Mix for qPCR (Applied Biosystems cat # A25741) and validated primer sets for K_Ca_3.1, K_Ca_2.3, eNOS, TRPV4 and GAPDH were obtained from Integrated DNA Technologies (see Table 1). PCR detection of select targets was performed using 1 ng of cDNA template per reaction organized in a MicroAmp™ Fast Optical 96-Well Reaction Plate with Barcode, 0.1 mL (Applied Biosystems cat # 4346906). Thermo-cycler amplification was performed with the QuantStudio 3 Real Time PCR instrument using the following settings: 50°C × 2 min, 95°C × 10 min, followed by 95°C × 16 s, and 60°C × 1 min steps repeated for 40 cycles. PCR specificity was checked by dissociation curve analysis. For each mRNA of interest, a standard curve was generated using known concentrations of cDNA (1, 2 and 4 ng) as a function of cycle threshold (CT). Control samples lacking cDNA template yielded no detectable fluorescence. Target gene expression in tissue-derived RNA samples was quantified relative to the expression of GAPDH using the following equation:

**TABLE 1:** Forward and reverse primers used in qPCR experiments.

	Forward primer	Reverse primer
K_Ca_3.1	5′-GAA​CTG​GCA​TCG​GAC​TCA​T-3′	5′-CAA​TAA​GAC​AAA​GGA​GGA​AGG​C-3′
K_Ca_2.3	5′-AGT​TTA​TCC​ACC​GTC​ATC​CTG-3′	5′-AGT​CAT​CTG​CAC​CAT​TGT​CG-3′
eNOS	5′-CTT​GAG​GAT​GTG​GCT​GTG​T-3′	5′-TGG​TCC​ACT​ATG​GTC​ACT​TTG-3′
TRPV4	5′-CTG​GAG​ATC​CTG​GTG​TAC​AAC-3′	5′-GAC​CAC​GTT​GAT​GTA​GAA​GGA​C-3′
GAPDH	5′-AAT​GGT​GAA​GGT​CGG​TGT​G-3′	5′-GTG​GAG​TCA​TAC​TGG​AAC​ATG​TAG-3′

Relative mRNA target expression = 2 ^ -(CT_target_–CT_GAPDH_), where CT = cycle threshold.

Reporting of q-PCR data has been performed in accordance with the MIQE guidelines.

### 2.10 Quantification and statistical analysis

Statistical analyses were performed with the GraphPad Prism Version 10 software (Dotmatics, San Diego, CA, Unites States). Data sets were analyzed using a one-way Analysis of Variance (ANOVA) test, and multiple comparisons were performed with a Tukey’s post-hoc test. A Student’s t-test was used to analyze potential differences within a given Apoe^−/−^ HFD treatment group, as noted in figure legends. Non-parametric data derived from qPCR measurements in abdominal aortic rings were analyzed by a Kruskal–Wallis one-way ANOVA and a Dunn’s post-hoc test. All data are presented as mean ± SD, and the number of animals analyzed under each protocol is indicated by N values stated in the figure captions. Differences were considered statistically significant when *P* < 0.05.

## 3 Results

### 3.1 Effects of *in vivo* administration of SKA-31 and senicapoc on abdominal aortic endothelial function in Apoe^−/−^ mice

**FIGURE 1:**
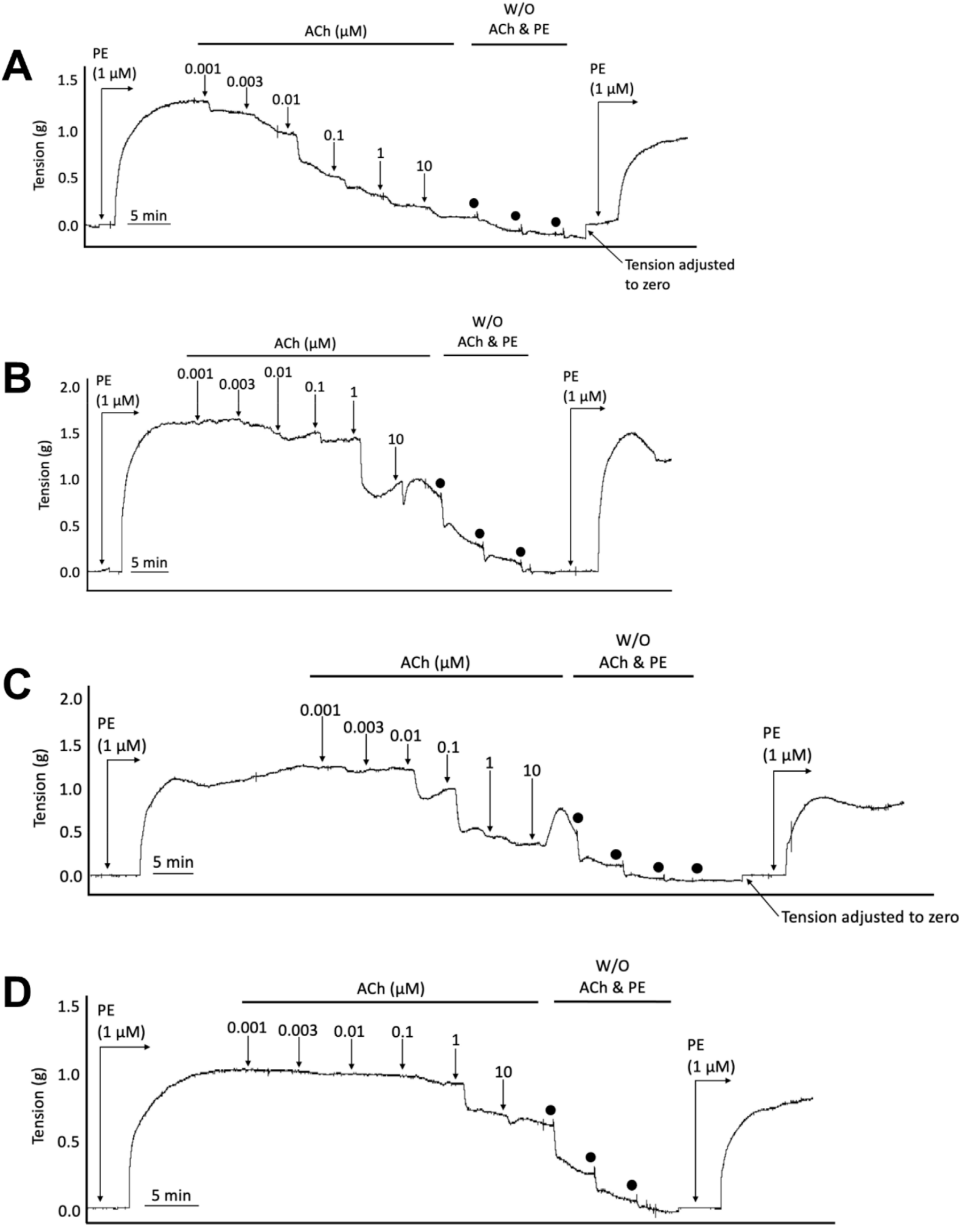
Representative wire myography tracings for cumulative concentration-dependent responses to acetylcholine (ACh) for abdominal aortic rings isolated from WT Normal Diet mice **(A)**, Apoe^−/−^ HFD vehicle-treated mice **(B)**, Apoe^−/−^ HFD SKA-31-treated mice **(C)**, and Apoe^−/−^ HFD senicapoc-treated mice **(D)**. Note that in the representative curves B, C, and D from Apoe^−/−^ mice, a modest contractile response to 10 µM acetylcholine can be seen after the initial relaxation response. W/O: washout of ACh and PE in the vessel bath by replacing the existing bath volume with fresh Krebs-Henseleit buffer. Individual washouts were indicated with black circles. Tension of the force transducer was adjusted to zero after a final washing step if the force recording registered <0 g. Note that 1 μM PE was added to the vessel bath at the end of the main protocol to verify that the acetylcholine-induced relaxation was reversible, and the aortic ring remained viable.

**FIGURE 2:**
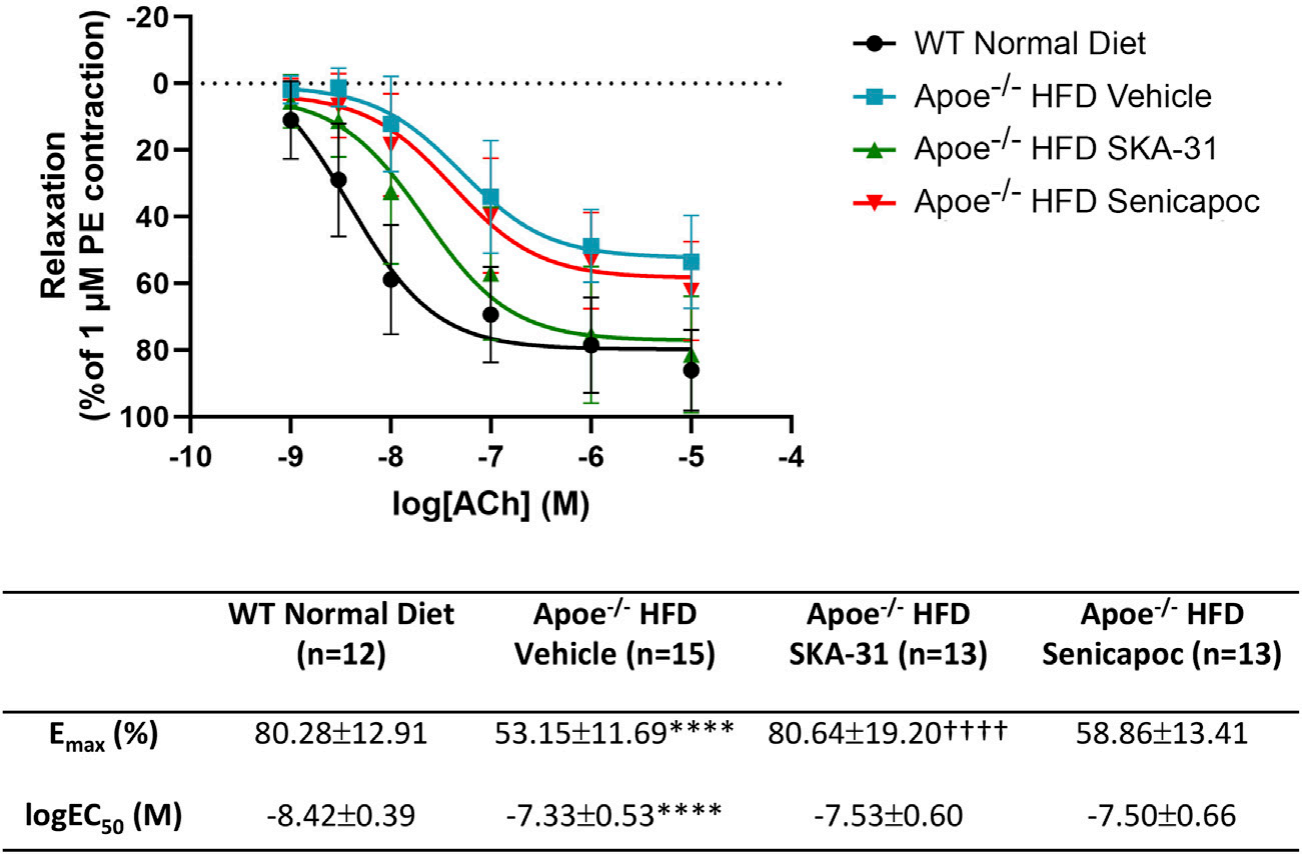
Acetylcholine-evoked endothelium-dependent relaxation responses in abdominal aortic rings isolated from WT mice and Apoe^−/−^ HFD mice treated with either vehicle or drug. Graph, concentration-response curves for increasing concentrations of acetylcholine (ACh) from 1 nM to 10 µM. Relaxation is reported as % decrease of phenylephrine (PE)-induced contraction (1 µM), with 100% relaxation being the point at which active tone returned to the pre-contraction level (i.e., in the absence of PE). Solid lines in the graph were generated by fitting data points with a non-linear regression equation, as described in the Materials and Methods section. Table, E_max_ and logEC_50_ values for the four experimental groups, which were obtained from the non-linear regression fits shown in panel A. The N values reported in the Table represent the number of individual animals within a given experimental group. Data are presented as mean ± SD and were analyzed with a one-way ANOVA, followed by a Tukey’s post-hoc test; ****P < 0.0001 vs. WT Normal Diet. ^††††^ *P* < 0.0001 vs. Apoe^−/−^ HFD Vehicle.

In Apoe^−/−^ mice, both conduit (e.g., thoracic aorta) and resistance (e.g., mesenteric) arteries display overt endothelial dysfunction, as evidenced by impairment of endothelium-dependent, acetylcholine-induced relaxation (d’Uscio et al., 2002; Crauwels et al., 2003). To examine if either SKA-31 or senicapoc treatment improved endothelial function in male Apoe^−/−^ mice maintained on a high fat diet (HFD) for 12 weeks, we measured acetylcholine-mediated relaxation in phenylephrine (PE) pre-constricted abdominal aortic rings by wire myography (Figure 1). In vehicle-treated Apoe^−/−^ HFD mice, both the maximal relaxation (E_max_) and potency (log EC_50_ value) of the acetylcholine (ACh) evoked response (i.e., 53.2% and −7.33 M, respectively) were significantly decreased compared with abdominal aortic rings from control WT mice on a regular chow diet (80.3% and −8.42 M, respectively; P < 0.0001) (Figure 2). In some instances, the severity of endothelial dysfunction in Apoe^−/−^ HFD mice promoted ACh-mediated aortic contraction at the highest concentration (Figures 1B, C). These observations are thus consistent with the presence of endothelial dysfunction in Apoe^−/−^ HFD mice (d’Uscio et al., 2001). Importantly, E_max_ for ACh-evoked relaxation was significantly improved in abdominal aortic rings from SKA-31-treated Apoe^−/−^ HFD mice (i.e., 80.6%) compared with the vehicle treatment group (P < 0.0001) (Figure 2). However, in senicapoc-treated Apoe^−/−^ HFD mice, E_max_ was not different than that observed in the vehicle-treated Apoe^−/−^ HFD mice (i.e., 58.9%) (Figure 2). Neither SKA-31 nor senicapoc treatments increased the potency of ACh to evoke relaxation in aortic rings compared with vehicle treated Apoe^−/−^ HFD mice (Figure 2).

### 3.2 Effects of SKA-31 and senicapoc treatments on smooth muscle-dependent relaxation in the abdominal aorta

**FIGURE 3:**
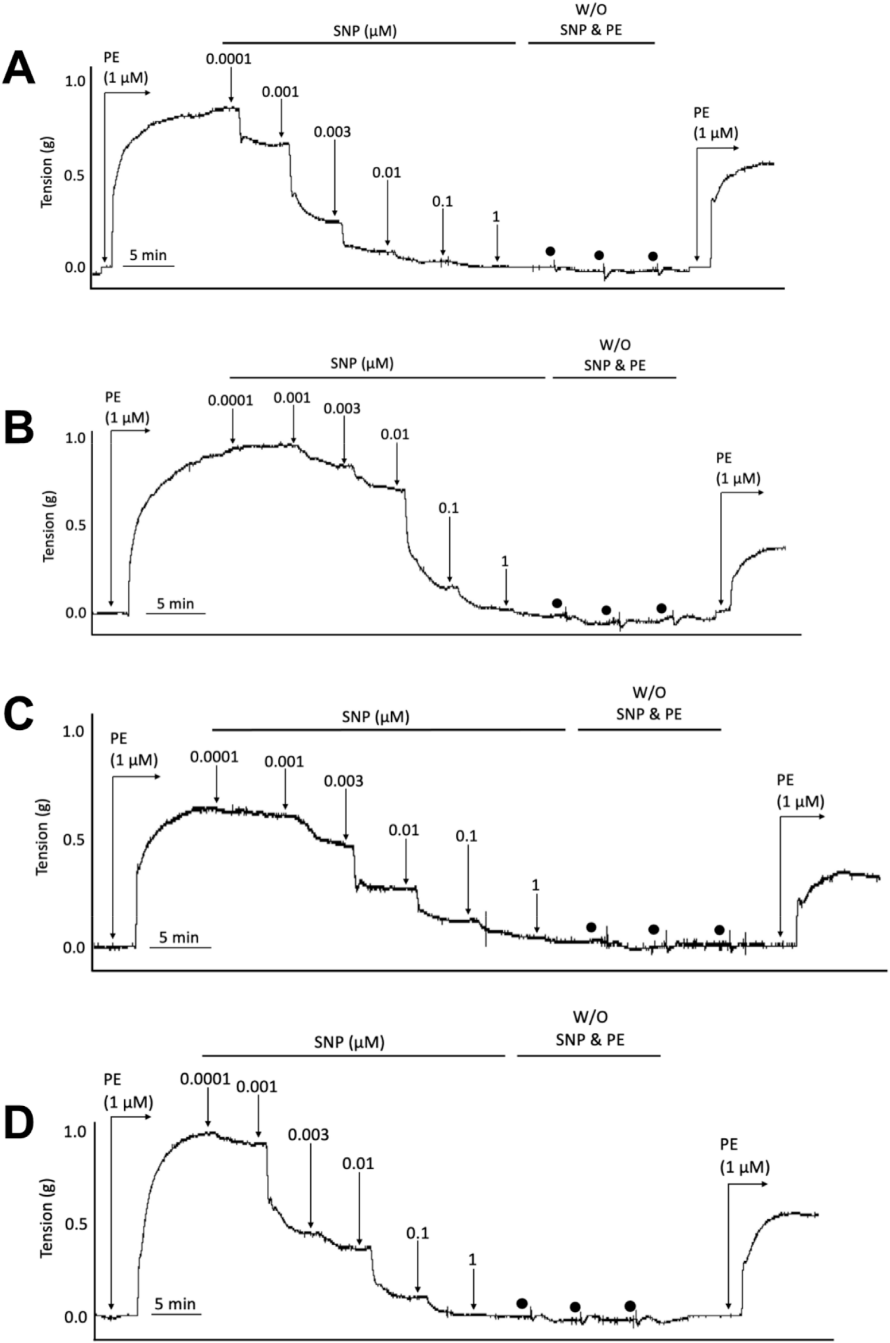
Representative wire myography tracings for cumulative concentration-dependent responses to sodium nitroprusside (SNP) for abdominal aortic rings isolated from WT Normal Diet mice **(A)**, Apoe^−/−^ HFD Vehicle-treated mice **(B)**, Apoe^−/−^ HFD SKA-31-treated mice **(C)**, and Apoe^−/−^ HFD senicapoc-treated mice **(D)**. W/O: washout of SNP and PE in the vessel bath by replacing the bath volume with fresh Krebs’ buffer. Individual washouts are indicated with black circles. Tension of the force transducer was re-adjusted to zero after a final washing step if the force recording was <0 g. Note that 1 μM PE was added to the vessel bath at the end of the main protocol to verify the reversibility of the SNP-induced relaxation.

**FIGURE 4:**
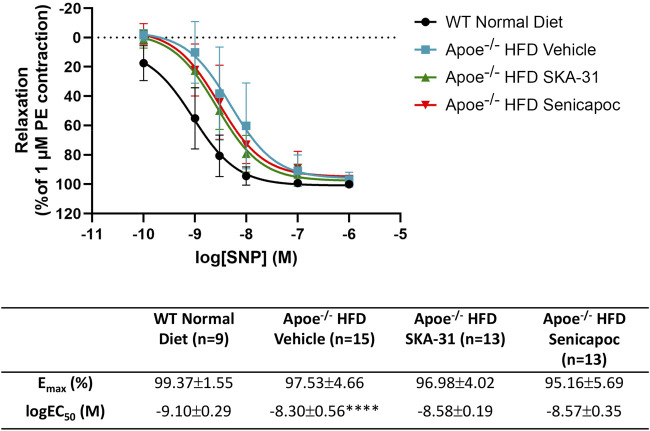
Sodium nitroprusside-evoked relaxation responses in abdominal aortic rings isolated from WT mice and Apoe^−/−^ HFD mice treated with either vehicle or drug. Graph, concentration-response curves for increasing concentrations of sodium nitroprusside (SNP) from 0.1 nM–1 µM. Relaxation is reported as the % reduction of phenylephrine (PE)-induced contraction (1 µM), with 100% relaxation being the point at which active tone returned to the baseline level (i.e., in the absence of PE). Solid lines in the graph were generated by fitting data points with a non-linear regression equation, as described in the Materials and Methods section. Table, E_max_ and logEC_50_ values for the four experimental groups, which were obtained from the non-linear regression fits displayed in panel A. Data were analyzed with a one-way ANOVA, followed by a Tukey’s post-hoc test and are presented as mean ± SD; ****P < 0.0001 vs. WT Normal Diet. The N values reported in the Table represent the number of individual animals within a given experimental group.

To determine whether SKA-31 or senicapoc administration affected vascular smooth muscle-dependent relaxation, we examined the vasoactive effects of sodium nitroprusside (SNP), an endothelium-independent vasodilator that acts directly on smooth muscle (McDonald and Murad, 1995; Ku, 1996). In abdominal aortic rings prepared from WT and Apoe^−/−^ HFD mice and pre-constricted with PE, SNP evoked a concentration-dependent relaxation that reached >90% of the initial PE constriction (Figure 3), and the observed E_max_ responses to SNP were not significantly different amongst the four experimental groups (Figure 4). SNP displayed higher potency in control WT aortic rings compared with rings from Apoe^−/−^ HFD vehicle-treated mice (log EC_50_ = −9.10 M vs. −8.30 M, respectively), but no significant differences were observed in either the log EC_50_ or E_max_ values for SNP amongst Apoe^−/−^ HFD mice treated with vehicle, SKA-31 or senicapoc (Figure 4).

### 3.3 Effects of SKA-31 and senicapoc treatments *in vivo* on phenylephrine-induced contraction of abdominal aorta *in situ*

**FIGURE 5:**
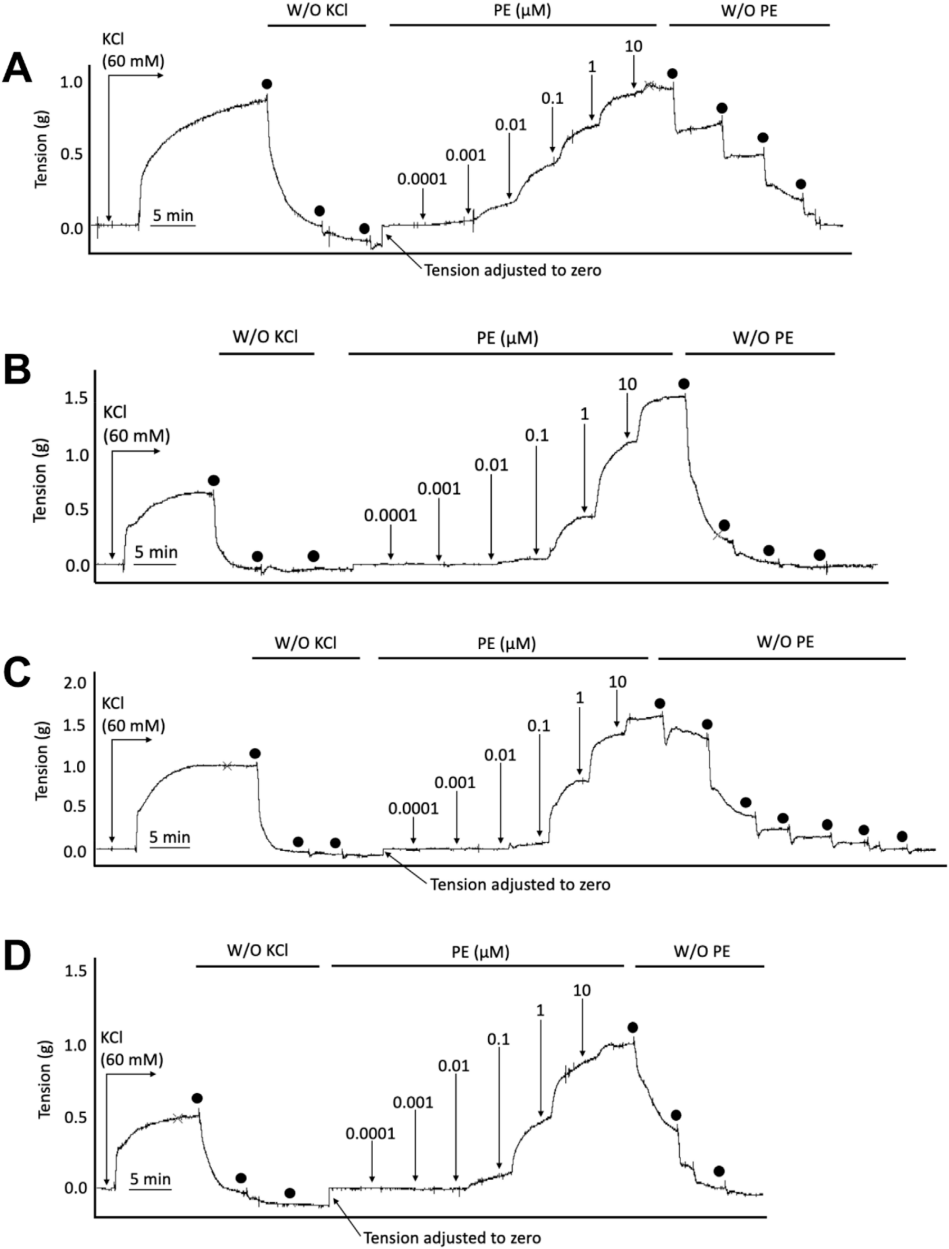
Representative wire myography tracings for cumulative concentration-dependent responses to phenylephrine (PE) for abdominal aortic rings isolated from WT Normal Diet mice **(A)**, Apoe^−/−^ HFD Vehicle-treated mice **(B)**, Apoe^−/−^ HFD SKA-31-treated mice **(C)**, and Apoe^−/−^ HFD senicapoc-treated mice **(D)**. W/O denotes washout of KCl and PE by replacing the bath volume with fresh Krebs’ buffer. Individual washouts are indicated with black circles. Tension of the force transducer was re-adjusted to zero after a final washing step if the force reading registered <0 g.

**FIGURE 6:**
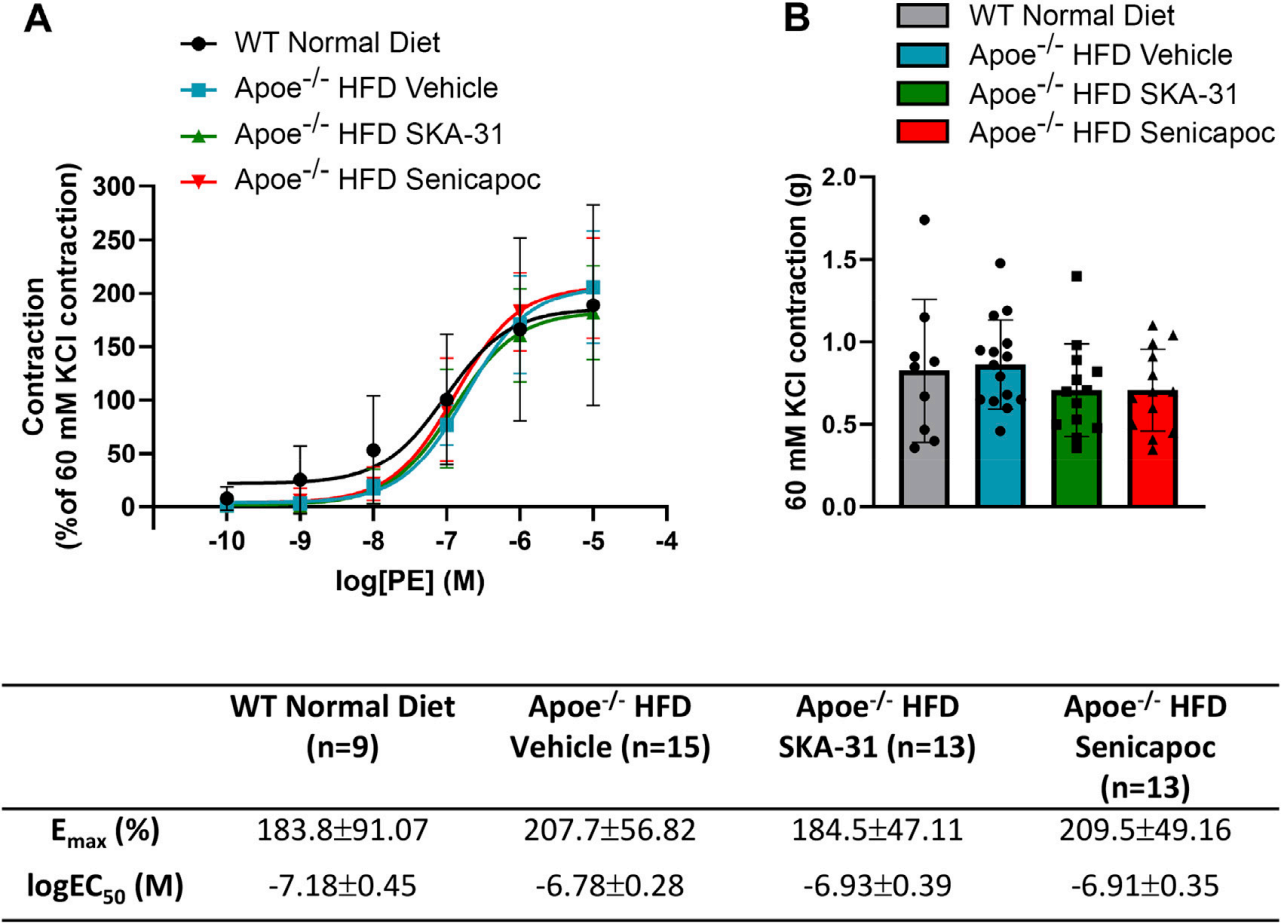
Phenylephrine-evoked contractile responses in abdominal aortic rings isolated from WT mice and Apoe^−/−^ HFD mice treated with either vehicle or drug. **(A)** Concentration-response curve for increasing concentrations of phenylephrine (PE) from 0.1 nM–10 µM. Contraction is reported as % of KCl-induced contraction (60 mM), with 100% contraction being the point at which PE-induced contraction matched the contraction induced by 60 mM KCl. Solid lines in the graph were generated by fitting data points with a non-linear regression equation, as described in the Materials and Methods section. **(B)** Maximal contraction to 60 mM KCl in the same abdominal aortic rings presented in A, with units in grams of force (g). Table, E_max_ and logEC_50_ values for the four experimental groups, which were obtained from the non-linear regression fits displayed in **(A)**. The N values reported in the Table represent the number of individual animals within a given experimental group. Data were analyzed with a one-way ANOVA, followed by a Tukey’s post-hoc test and are presented as mean ± SD. No statistically significant differences were observed.

Earlier data have shown that thoracic aortic rings from Apoe^−/−^ mice can exhibit a more robust contractile response to phenylephrine (PE) compared with age and sex-matched WT mice (Fransen et al., 2008). We thus examined if SKA-31 or senicapoc treatment altered either the maximal response or sensitivity to PE utilizing abdominal aortic rings isolated from vehicle, SKA-31 and senicapoc treated Apoe^−/−^ HFD mice. Figure 5 shows representative tracings of concentration-dependent contraction induced by PE in aortic rings from control WT and Apoe^−/−^ HFD mice treated *in vivo*. Following normalization of PE-induced responses to the steady-state contraction evoked by bath-applied 60 mM KCl, we observed no significant differences in the maximal level of contraction (E_max_) evoked by PE amongst the four experimental groups (Figure 6A). Additionally, the potency of PE evoked contraction (i.e., log EC_50_ value) in abdominal aortic rings from Apoe^−/−^ HFD vehicle-treated mice rings was not greater than that observed in aortic rings from the control WT mice (*P* = 0.063), and no significant differences were observed among the log EC_50_ values for PE in abdominal aortic rings from vehicle, SKA-31 and senicapoc-treated Apoe^−/−^ HFD mice (*P* > 0.70) (Figure 6A). Finally, the absolute magnitude of contraction/tension generated following exposure of abdominal aortic rings to external 60 mM KCl did not differ among the control and Apoe^−/−^ HFD mice (Figure 6B).

### 3.4 Effects of SKA-31 and senicapoc on expression of select mRNA targets in abdominal aorta

**FIGURE 7:**
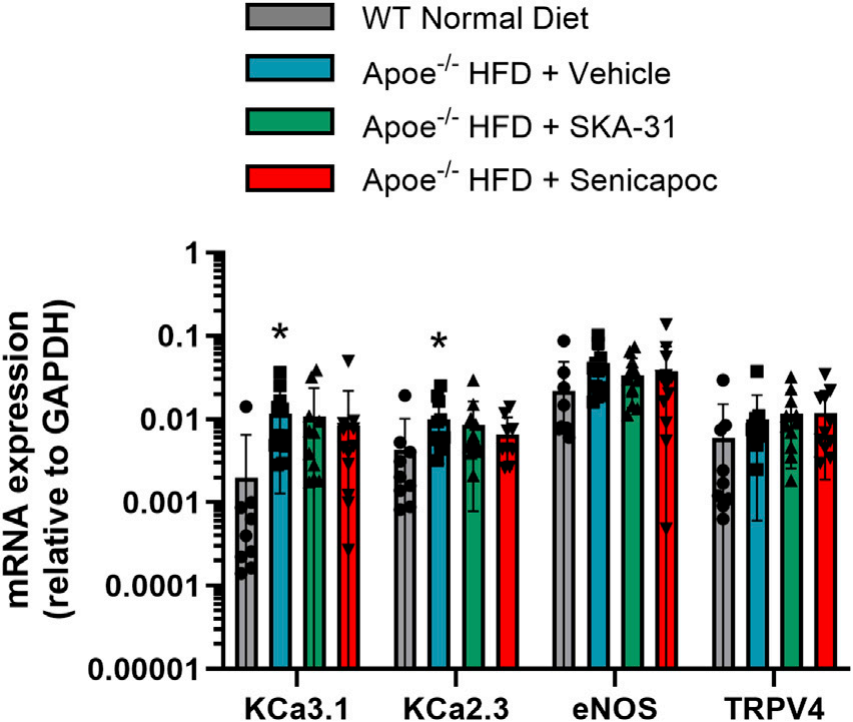
Investigation of key endothelial signaling molecules in abdominal aorta by q-PCR analyses. The mRNA levels of the selected molecular targets are plotted relative to the mRNA expression of GAPDH (internal reference). Plotted symbols represent individual animals in the wild-type (WT) normal diet group (n = 9) and Apoe^−/−^ HFD mice treated with vehicle (n = 11–12), 10 mg/kg SKA-31 (n = 11–12) or 40 mg/kg senicapoc (n = 9–12). The asterisk (*) indicates a statistical difference between the WT normal diet mice and Apoe^−/−^ + Vehicle treated mice (P < 0.02). No differences in the expression level of a given mRNA target were noted among the 3 groups of treated Apoe^−/−^ HFD mice (P > 0.2, Kruskal–Wallis one-way ANOVA and Dunn’s post-hoc test).

To examine whether SKA-31 or senicapoc treatment altered the endothelial expression of key signaling proteins linked to stimulus-evoked relaxation, select mRNA targets (i.e., eNOS, K_Ca_3.1, K_Ca_2.3 and TRPV4 channels) were quantified by quantitative PCR in abdominal aortic rings. Due to the limited tissue available for RNA extraction, it was not possible to interrogate additional targets. Figure 7 shows that the level of expression of these select mRNA targets was unaffected in Apoe^−/−^ HFD mice by 12-week administration of either SKA-31 or senicapoc compared with vehicle treatment, suggesting that the observed augmentation of ACh-induced relaxation by SKA-31 treatment is likely not due to modified expression of these signaling molecules in the abdominal aortic wall. Prolonged treatment with the K_Ca_3.1 blocker TRAM-34 also did not modify mRNA or protein expression of the endothelial K_Ca_3.1 channel in Apoe^−/−^ atherosclerotic mice (Toyama et al., 2008).

### 3.5 Effects of SKA-31 and senicapoc treatments on fatty plaque area in the thoracic aorta and aortic sinus of Apoe^−/−^ HFD mice

**FIGURE 8:**
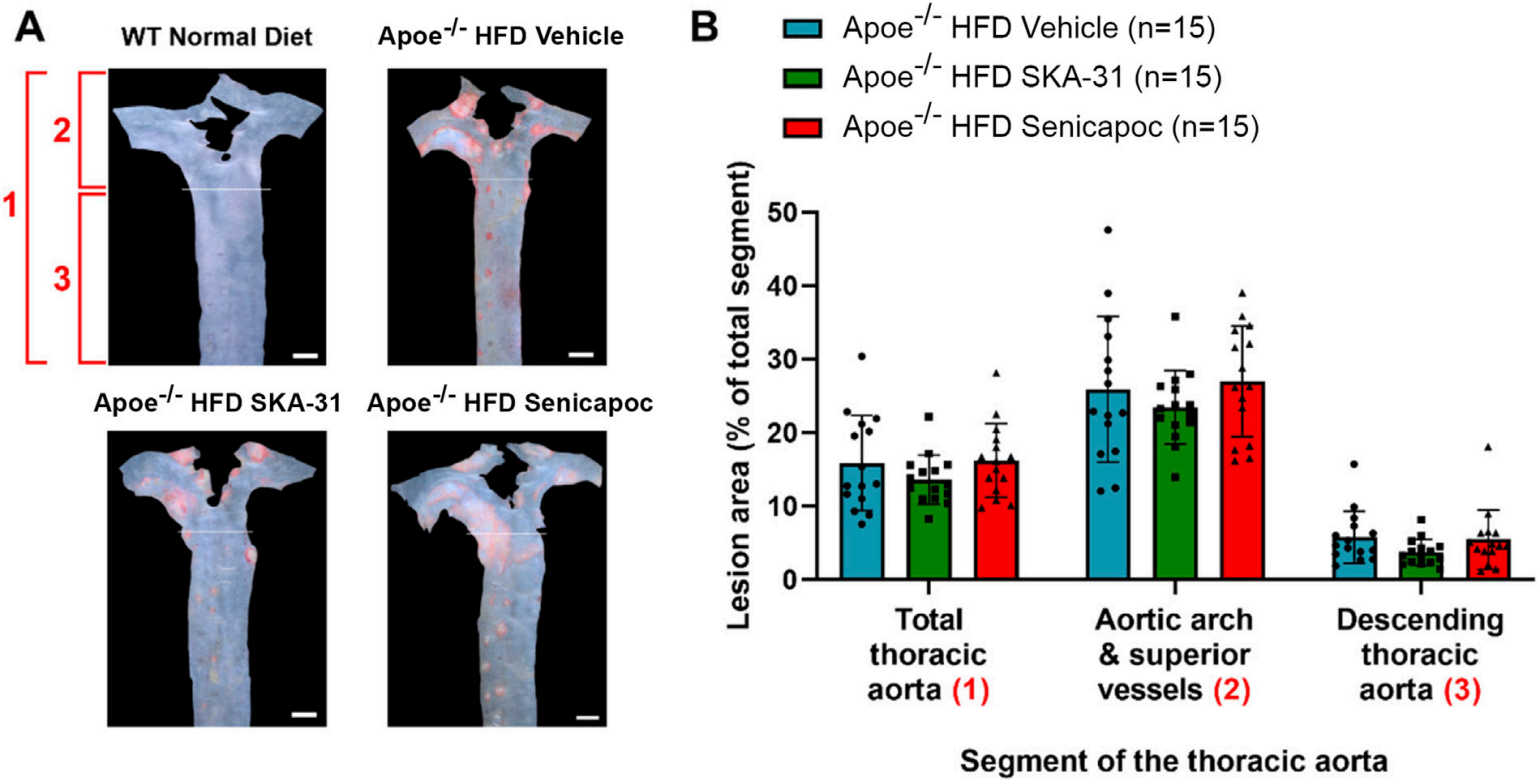
Fatty plaque measurement in the thoracic aorta from WT and Apoe^−/−^ HFD mice. **(A)** Representative *en face* images of the thoracic aorta from mice in the four experimental groups. The area labelled (1) encompasses the total thoracic aorta (i.e., the areas of (2) + (3) combined). The area labelled (2) consists of the aortic arch and superior vessels. The area labelled (3) consists of the descending thoracic aorta. WT Normal Diet aortae showed very little Oil Red O staining for fatty plaques (red staining made up <1% of total thoracic aorta area) and were excluded from lesion area analyses. Scale bar displayed in the lower righthand corner of images in panel A represents 1 mm. **(B)** Quantification of lesion area, shown as % of the aortic area covered by lesions. Lesion areas were analyzed with a one-way ANOVA, followed by a Tukey’s post-hoc test; no statistically significant differences were observed. Data are presented as mean ± SD and N values are stated in the panel legend.

**FIGURE 9:**
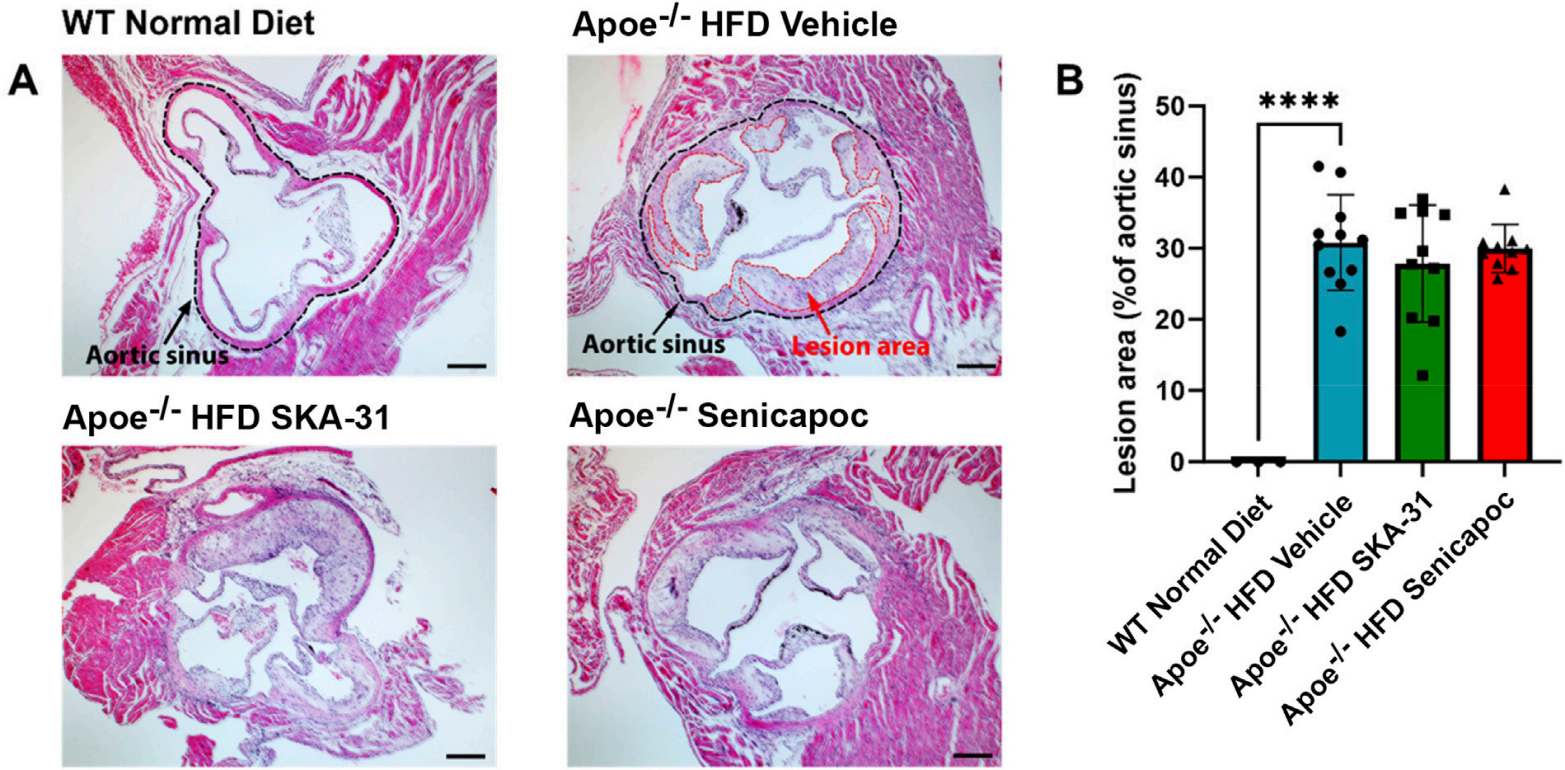
Lesion area measurement in the aortic sinuses of WT and Apoe^−/−^ HFD mice. **(A)** Representative H&E-stained aortic sinus sections from WT Normal Diet mice (n = 3), Apoe^−/−^ HFD Vehicle-treated mice (n = 9), Apoe^−/−^ HFD SKA-31-treated mice (n = 11), and Apoe^−/−^ HFD senicapoc-treated mice (n = 9). In representative images for WT Normal Diet and Apoe^−/−^ HFD vehicle treated mice, the border of the aortic sinus is outlined in black, and lesion areas inside the aortic sinus are outlined in red. The scale bar displayed in the lower righthand corner of images represents 0.2 mm. **(B)** Lesion area quantified as a percentage of aortic sinus area. Data in panel B are presented as mean ± SD, and were analyzed with a one-way ANOVA, followed by a Tukey’s post-hoc test; ****P < 0.0001.

Endothelial dysfunction is an important event that precedes and promotes atherosclerotic fatty plaque development (Davignon and Ganz, 2004). As prolonged SKA-31 administration improved endothelium-dependent vasorelaxation (Figure 2), we investigated whether this treatment secondarily affected the extent of atherosclerotic lesions present in the thoracic aorta, a region of prominent fatty plaque development in Apoe^−/−^ mice (Nakashima et al., 1994; Toyama et al., 2008; Lin et al., 2015). Regardless of whether the entire thoracic aorta or subregions of the thoracic aorta were examined (i.e., aortic arch and superior vessels, descending thoracic aorta), we did not observe differences in the extent of atherosclerotic lesion area in SKA-31 and senicapoc treated Apoe^−/−^ HFD mice compared with Apoe^−/−^ HFD mice treated with vehicle (Figure 8). We also examined the aortic sinus as another region of prominent lesion development in Apoe^−/−^ mice and one which may develop plaques earlier than the thoracic aorta (Meir and Leitersdorf, 2004). Although fatty plaques were prominent in the aortic sinus of vehicle-treated Apoe^−/−^ HFD mice (P < 0.0001 compared with control WT mice), neither SKA-31 nor senicapoc treatment *in vivo* modified the extent of fatty plaque area in the aortic sinus (Figure 9).

**FIGURE 10:**
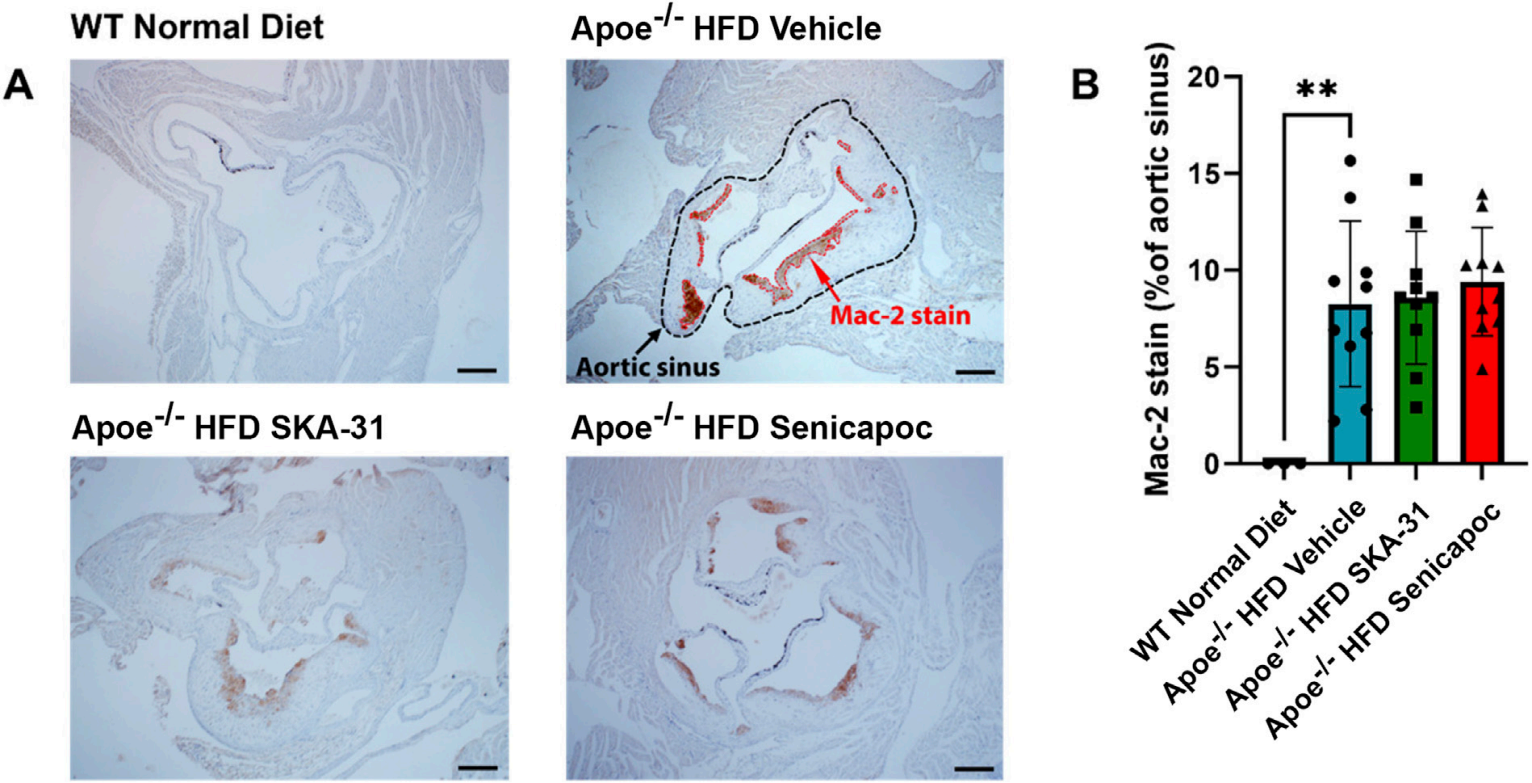
Macrophage content in the aortic sinuses of WT and Apoe^−/−^ HFD mice. **(A)** Representative anti-Mac-2 immuno-stained aortic sinus sections from WT Normal Diet (n = 3), Apoe^−/−^ HFD Vehicle-treated (n = 10), Apoe^−/−^ HFD SKA-31-treated (n = 10), and Apoe^−/−^ HFD senicapoc-treated (n = 10) mice. The Apoe^−/−^ HFD Vehicle representative image shows a black outline representing the border of the aortic sinus, while the anti-Mac-2-stained areas within the aortic sinus are outlined in red. The scale bar displayed in the lower righthand corner of each image in **(A)** represents 0.2 mm. **(B)** Mac-2-stained area plotted as a percentage of the visible aortic sinus area. Data in **(B)** are presented as mean ± SD and were analyzed with a one-way ANOVA, followed by a Tukey’s post-hoc test; **P < 0.01.

**FIGURE 11:**
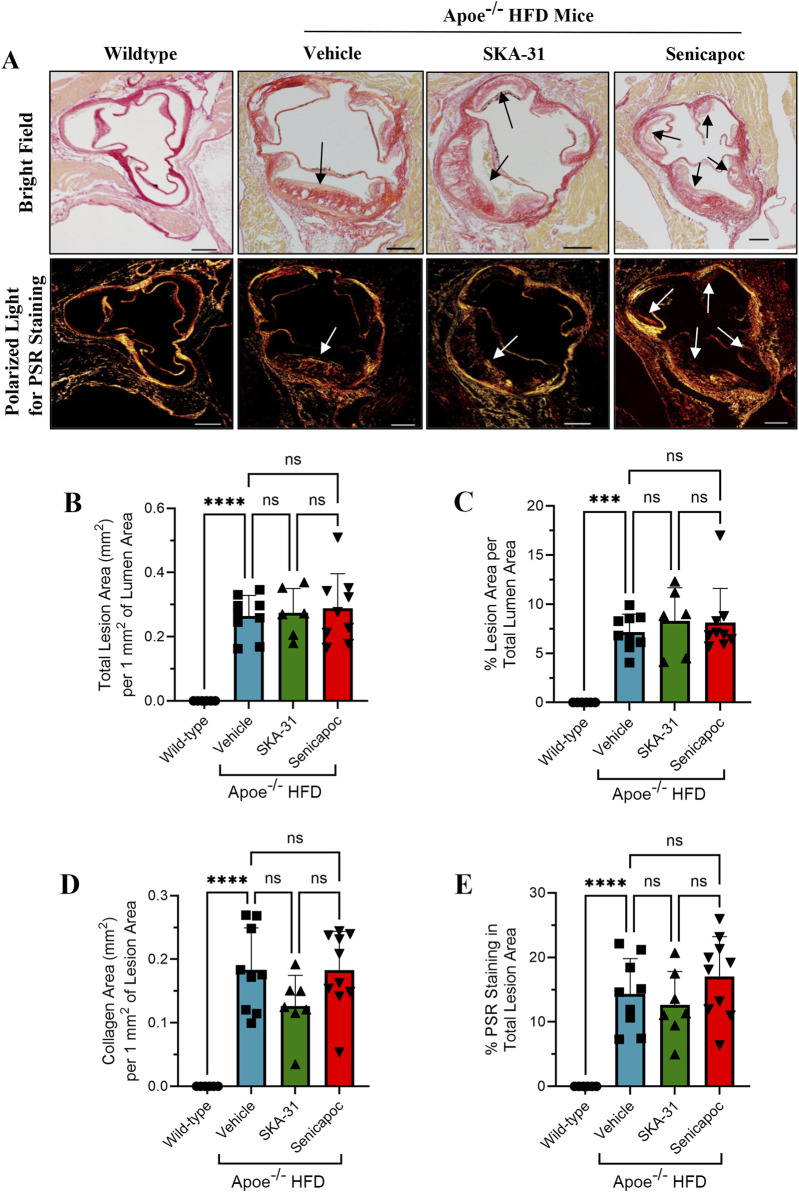
Detection and quantification of collagen staining in the aortic sinus. **(A)** shows representative thin histological sections of fixed aortic sinus from wild-type mice (n = 5) and Apoe^−/−^ mice on a high fat diet (HFD) treated with either drug vehicle (n = 9), 10 mg/kg SKA-31 (n = 6–7) or 40 mg/kg senicapoc (n = 9–10). The upper row displays aortic sinus sections viewed under brightfield illumination using differential interference contrast (DIC) filtering, while the lower row shows adjacent sections from the same heart stained with Picrosirius Red (PSR) and illuminated with a linear polarized lens and captured with DIC filtering. All aortic sinus sections displayed in **(A)** were recorded using a ×4 objective. Brightfield illumination was used to quantify the total lesion area in mm^2^ per 1 mm^2^ of aortic lumen area **(B)**, and the percent of total lumen area occupied by atherosclerotic lesion **(C)**. Arrows in the upper and lower images in panel A indicate plaque-like lesions protruding into the aortic lumen. The amount of collagen detected per mm^2^ of atherosclerotic lesion is displayed in **(D)** and the percentage of PSR staining observed in total lesion areas is plotted in **(E)**. The scale bar shown in the lower righthand corner of images in **(A)** represents 100 μm. Statistical analyses were performed using one-way ANOVA and a Tukey’s post-hoc test; ***P < 0.001, ****P < 0.0001.

**FIGURE 12:**
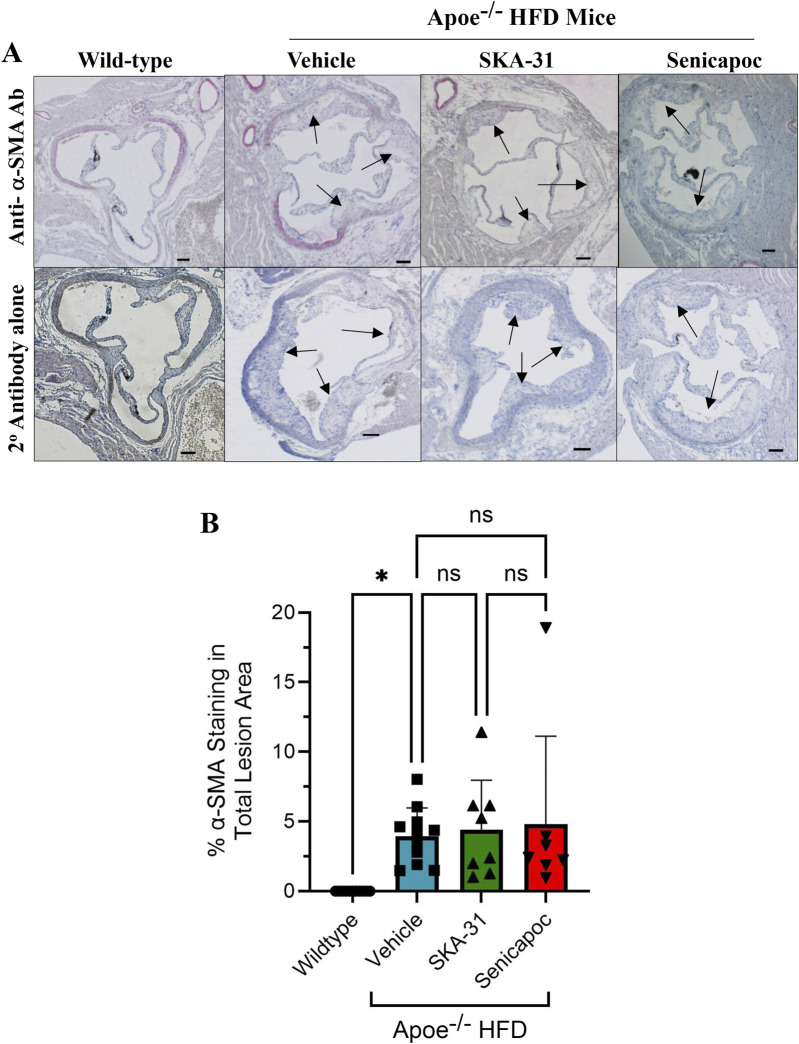
Detection and quantification of alpha-smooth muscle actin (α-SMA) staining in the aortic sinus. **(A)** shows representative thin histological sections of immune-stained aortic sinus from wild-type (WT) mice (n = 7) and Apoe^−/−^ HFD mice treated with drug vehicle (n = 11), 10 mg/kg SKA-31 (n = 8) or 40 mg/kg senicapoc (n = 7). The upper row displays aortic sinus sections immuno-stained with a rat anti-mouse α-SMA antibody followed by staining with a biotinylated rabbit anti-rat secondary antibody, while the lower row shows adjacent sections from the same heart stained with the biotinylated rabbit secondary antibody alone. Black arrows indicate plaque-like lesions protruding into the aortic lumen. All aortic sinus sections displayed in panel F were recorded using a ×10 objective. Brightfield illumination was used to quantify the percent of total lesion area exhibiting α-SMA staining; these data are quantified in panel **(B)**. The scale bar shown in the lower righthand corner of images in **(A)** represents 200 μm. Statistical analyses were performed using one-way ANOVA and a Tukey’s post-hoc test; *P < 0.05.

Further characterization of atherosclerotic lesions revealed the presence of inflammatory-type macrophages in the aortic sinus of Apoe^−/−^ HFD mice, a reported marker of atherosclerosis severity (Gui et al., 2022). Immunohistochemical staining with the Mac-2 antibody revealed substantive immunoreactivity, with no detectable staining in control WT mice (Figure 10). Neither SKA-31 nor senicapoc treatment altered the degree of Mac-2 immuno-staining in the aortic sinus compared with vehicle treated Apoe^−/−^ HFD mice. Plaques in the aortic sinus region also exhibited substantive staining of collagen and alpha-smooth muscle actin (α-SMA) (Figures 11, 12), which are reported to influence plaque stability and rupture (Adiguzel et al., 2009; Allahverdian et al., 2018). The levels of these two markers in Apoe^−/−^ HFD mice were unchanged by either SKA-31 or senicapoc administration (Figures 11, 12). Collectively, these observations indicate that SKA-31 and senicapoc treatments *in vivo* did not significantly alter the extent of fatty plaque area in the thoracic aorta and aortic sinus of Apoe^−/−^ HFD mice, the presence of macrophages or the content of collagen and α-SMA staining in atherosclerotic lesions located within the aortic sinus region.

Despite the presence of atherosclerotic plaques in the thoracic aorta and aortic sinus, the magnitude of pulse wave velocity (PWV) measured *in vivo* in Apoe^−/−^ HFD mice was similar to that observed in control WT mice (Supplementary Table S1) and was unchanged by administration of either SKA-31 or senicapoc. In addition, the magnitudes of left ventricular ejection fraction, fractional shortening and cardiac output in vehicle-treated Apoe^−/−^ HFD mice were similar to those observed in WT controls, although end-systolic and end-diastolic left ventricular volumes, along with stroke volume were significantly decreased in the Apoe^−/−^ HFD mice (Supplementary Table S2). None of the cardiac functional parameters observed in Apoe^−/−^ HFD were modified by SKA-31 or senicapoc treatment.

### 3.6 Effects of SKA-31 and senicapoc treatments on serum lipid levels and cytokines, body weight and length, and organ morphometrics

Prolonged administration of either SKA-31 or senicapoc did not significantly affect longitudinal gain in body weight or body length in Apoe^−/−^ HFD mice compared with vehicle treated animals (Supplementary Figure S1). As expected, serum lipid levels (i.e., total cholesterol, high-density lipoprotein cholesterol and low-density lipoprotein cholesterol) were substantially elevated in vehicle treated Apoe^−/−^ HFD mice compared with control WT mice (Supplementary Figure S2) but were not significantly affected by either SKA-31 or senicapoc treatment. However, a modest increase in serum triglyceride was observed in Apoe^−/−^ HFD mice treated with SKA-31 (*P* < 0.05) (Supplementary Figure S2B). Compared with WT mice, vehicle-treated Apoe^−/−^ HFD mice exhibited elevated serum concentrations of pro-inflammatory cytokines (e.g., GM-CSF, interferon-gamma, interleukin-2) (Supplementary Figure S3); in SKA-31 treated Apoe^−/−^ HFD mice, the serum level of IFN-γ was significantly reduced vs. vehicle treatment.

Histological analyses of the heart (Supplementary Figure S4), brain hippocampus (Supplementary Figure S5), brain cerebellum (Supplementary Figure S6), liver parenchyma (Supplementary Figure S7), and the renal medulla (Supplementary Figure S8) did not reveal adverse morphological changes in these tissues following prolonged treatment with either SKA-31 or senicapoc. Interestingly, Apoe^−/−^ HFD mice exhibited a reduced renal corpuscle area compared with control WT mice, which was restored to the control level by senicapoc treatment (Supplementary Figure S8B). Collectively, these observations suggest that both SKA-31 and senicapoc treatments were well tolerated by Apoe^−/−^ HFD mice over the 12-weeks of daily administration.

## 4 Discussion

We have previously reported that acute treatment of isolated resistance arteries from T2D rats and humans with the selective K_Ca_ channel activator SKA-31 reverses endothelial dysfunction (Mishra et al., 2021), and that prolonged *in vivo* administration of SKA-31 mitigates endothelial dysfunction in isolated mesenteric arteries from aged rats (Mathew John et al., 2020). SKA-31 administration further restores flow-mediated dilation in the femoral artery of T2D rats *in vivo* (Mishra et al., 2024). As endothelial dysfunction is considered an early causative event in the development of atherosclerosis (Davignon and Ganz, 2004), we sought to determine whether enhancement of endothelial K_Ca_2.3/K_Ca_3.1 channel activity *in vivo* would ameliorate endothelial dysfunction in the aorta of atherosclerotic Apoe^−/−^ mice maintained on a high fat diet (HFD). In the present study, we found that vehicle treated Apoe^−/−^ HFD mice exhibited impaired endothelium-dependent relaxation of PE-induced tone in isolated abdominal aortic segments compared with sex- and age-matched WT mice, whereas daily administration of 10 mg/kg SKA-31 starting at 8 weeks of age mitigated this endothelial deficit (Figures 1, 2). This improvement in aortic endothelial function induced by SKA-31 did not occur secondarily to changes in aortic smooth muscle activity, as neither the magnitude nor sensitivity of abdominal aortic ring relaxation by the direct smooth muscle vasorelaxant SNP (Figure 4), or the actions of the smooth muscle vasoconstrictor PE (Figure 6) were modified following *in vivo* SKA-31 administration. The observed SKA-31 induced improvement in aortic endothelial function was also not associated with increased mRNA expression of eNOS and KCa3.1, KCa2.3 and TRPV4 channels that contribute to endothelium-dependent vasodilatory signaling (Figure 7) (Köhler et al., 2016; Vanhoutte et al., 2017; Chen and Sonkusare, 2020). Mechanistically, endothelium-dependent relaxation in the aorta of WT and Apoe^−/−^ HFD mice is due almost exclusively to NO signaling (d’Uscio et al., 2001; Balarini et al., 2013). As SKA-31 administration did not modify the sensitivity of aortic smooth muscle to the vasorelaxant NO donor SNP or the vasocontractile action of PE in Apoe^−/−^ HFD mice (Figures 4, 6A), we speculate that SKA-31 treatment may have increased the efficacy of endothelial eNOS activation by ACh. We have previously reported that enhancement of endothelial KCa channel activity can improve activation of eNOS by calcium-mobilizing agonists (Sheng et al., 2009), along with endothelial Ca^2+^ mobilization (Mishra et al., 2021), and such processes may contribute to our observed effects in aortic rings from SKA-31 treated Apoe ^−/−^ HFD mice.

In contrast to the observed improvement in aortic vasoreactivity, treatment of Apoe^−/−^ HFD mice with SKA-31 did not decrease the area of fatty plaque formation in the thoracic aorta (Figure 7) or aortic sinus (Figure 8). We further observed that the presence of atherosclerotic plaques was not associated with impairment of either aortic pulse wave velocity (Supplementary Table S1) or myocardial contractility (e.g., ejection fraction, fractional shortening, cardiac output) (Supplementary Table S2) or the presence of structural cardiac hypertrophy (Supplementary Figure S4). These observations in ∼20-week old, vehicle treated Apoe^−/−^ HFD mice thus indicate a temporal delay between the early appearance of atherosclerotic-like lesions in the proximal and thoracic aorta and functional deficits in cardiovascular (CV) parameters that are common sequelae of such lesions. As endothelial dysfunction typically precedes the development and progression of atherosclerosis, we speculate that the observed improvement of endothelial function in Apoe^−/−^ HFD mice by SKA-31 treatment may oppose the functional impairments of CV performance that develop beyond the early stage of atherosclerosis when plaques become more permanent and progressive, rather than directly reduce plaque formation. This speculation thus aligns with the temporal relation between progressive plaque formation and the delayed appearance of functional deficits reported in atherosclerotic subjects (Bentzon et al., 2014). Related to these findings, clinical data indicate that treatment of atherosclerosis with statin-type drugs decreases both fatty plaque area (i.e., plaque regression) and the rupture of established plaques, thereby reducing the risk for subsequent CV events such as stroke and myocardial infarction (Weissberg, 2000; Bittencourt and Cerci, 2015). As statin drugs are known to improve endothelial function in atherosclerotic arteries (Wolfrum et al., 2003; Ii and Losordo, 2007), this action also likely contributes to their anti-atherosclerotic effects and could be mimicked in part by the *in vivo* administration of an endothelial K_Ca_ channel activator that mitigates aortic endothelial dysfunction. SKA-31 treatment did not significantly alter the content of resident macrophages, collagen and α-SMA associated within visible atherosclerotic lesions in the aortic sinus of Apoe^−/−^ HFD mice (Figures 10–12), suggesting that plaque stability was not altered. Of relevance, Bi and colleagues have reported that treatment of quiescent human coronary VSMCs with SKA-31 inhibited the de-differentiation of these cells and their proliferation in response to mitogenic stimulation by PDGF (i.e., Platelet-Derived Growth Factor) (Bi et al., 2013). These latter findings suggest that SKA-31 treatment may further reduce the risk of plaque rupture by stabilizing VSMC phenotype.

In contrast to SKA-31 treatment, daily administration of the K_Ca_3.1 channel blocker senicapoc (40 mg/kg) did not mitigate endothelial dysfunction in abdominal aortic rings compared with vehicle treated Apoe^−/−^ HFD mice (Figure 2), nor did it modify SNP-mediated relaxation (Figure 4), or PE-induced smooth muscle contraction (Figure 6). Previous reports have described reduced plaque formation and lesion-associated macrophages in Apoe^−/−^ HFD mice following daily administration of TRAM-34, another small molecule K_Ca_3.1 channel inhibitor (Toyama et al., 2008; Tharp and Bowles, 2021; Jiang et al., 2022). However, these earlier studies did not examine endothelium-dependent relaxation or aortic vascular reactivity in TRAM-34 treated Apoe^−/−^ HFD mice, so it is unclear if the observed reduction in atherosclerotic lesion area improved aortic endothelial function.

Somewhat surprisingly, senicapoc treatment did not significantly decrease fatty plaque area in the thoracic aorta (Figure 7) or the aortic sinus (Figure 8) or alter macrophage accumulation in the aortic sinus (Figure 10), in contrast to observations reported for TRAM-34 administration to Apoe^−/−^ HFD mice (Toyama et al., 2008). As TRAM-34 and senicapoc have a similar IC_50_ for KCa3.1 inhibition (20 nM and 11 nM, respectively), we selected senicapoc instead of the tool compound TRAM-34 because senicapoc is orally availability and has been approved for use in human clinical trials (Ataga et al., 2011; Ataga et al., 2021). In our study, 7 out of 10 Apoe^−/−^ HFD mice treated daily with 40 mg/kg senicapoc exhibited a steady-state plasma concentration of drug (166 ± 128 nM) 16–20 h post administration, whereas the plasma level of senicapoc in 3 animals was below detection. These observations confirm that senicapoc was available in the blood of Apoe^−/−^ HFD mice treated with 40 mg/kg but may have been sub-optimally dosed compared with TRAM-34 at 120 mg/kg in previous studies (Toyama et al., 2008; Tharp and Bowles, 2021; Jiang et al., 2022). Senicapoc, which has 12 days half-live in humans, has a short (∼1-h) half-life in mice and unlike the more lipophilic TRAM-34, does not accumulate in adipose tissue. In contrast, mice dosed with a higher amount of TRAM-34 (e.g., 100 mg/kg or more) exhibited a long-lasting drug depot in fat (Toyama et al., 2008). Given this difference in pharmacokinetic profile, use of a more aggressive dosing regimen for senicapoc (e.g., 120 mg/kg daily) may have led to more robust effects of this compound on atherosclerotic plaque formation in our Apoe^−/−^ HFD mice.

Along with the data set, another important consideration of our study are the potential limitations. As described, the Apoe^−/−^ mice utilized in our project were young, male animals (i.e., ∼20 weeks of age at protocol termination); thus, it is uncertain whether the observed effects of SKA-31 on endothelial function apply to females or will be evident in the atherosclerotic arteries of aged mice that approximate the age of atherosclerotic humans. Extending the duration of our study could have allowed the secondary effects of atherosclerosis to become more prominent in our Apoe^−/−^ HFD mice (e.g., increased aortic stiffening, myocardial damage and impaired contractility), and provided greater opportunity for SKA-31 mediated improvements in endothelial function to oppose such deficits. Our experimental design further focused on SKA-31 treatment primarily as a preventative strategy for endothelial dysfunction, but we did not examine the potential efficacy of this strategy to reverse established dysfunction and its related CV consequences. Our study also did not explicitly examine the individual contributions of endothelial K_Ca_3.1 and K_Ca_2.3 channels to the observed enhancement of aortic endothelium-dependent relaxation. Although SKA-31 acts on both K_Ca_2.3 and K_Ca_3.1 channels, it exhibits ∼10-fold higher potency for K_Ca_3.1 vs. K_Ca_2.3 channels (EC_50_ ∼0.3 μM vs. 2 μM, respectively) (Sankaranarayanan et al., 2009) and would thus be expected to enhance K_Ca_3.1 channel activity more effectively when administered *in vivo*. Given this pharmacological bias, it is reasonable to suggest that K_Ca_2.3 activity contributes only modestly to the observed effects of SKA-31 administration in Apoe^−/−^ HFD mice. Furthermore, prior studies have reported that K_Ca_3.1 inhibition reduces the severity of atherogenesis in Apoe^−/−^ HFD mice, likely by decreasing inflammatory events and vascular smooth muscle de-differentiation and proliferation (Toyama et al., 2008; Tharp and Bowles, 2021; Xu et al., 2017; Bi et al., 2013). These observations thus provide additional rationale for focusing on K_Ca_3.1 vs. K_Ca_2.3 activity. At the molecular level, the limited availability of abdominal aortic tissue impacted our ability to perform Western blot analysis to complement the q-PCR data presented in Figure 7. Finally, our measurements of fatty plaque formation and vascular reactivity were taken from adjacent, but separate segments of the aortic tube (i.e., thoracic and abdominal aorta, respectively). This strategy was designed to maximize the use of limited aortic tissue available for both types of measurements and is supported in part by observations that the abdominal aortae of Apoe^−/−^ mice exhibit fatty plaque-like lesions comparable to those present in the thoracic aorta (Nakashima et al., 1994; Toyama et al., 2008; Lin et al., 2015). In addition, endothelial dysfunction has been described in the thoracic aorta of Apoe^−/−^ mice (Crauwels et al., 2003), and we are unaware of reports suggesting that endothelial cell phenotype and function in the thoracic aorta substantially differ from those found in the abdominal segment. K_Ca_2.3 and K_Ca_3.1 channels are expressed throughout the aortic endothelium (Si et al., 2006; Choi et al., 2016), suggesting that SKA-31 would be pharmacologically effective in both the thoracic and abdominal aorta. It is further conceivable that examination of other vascular beds in Apoe^−/−^ HFD + SKA-31 treated mice (e.g., coronary, mesenteric, skeletal muscle) may have revealed broader effects of SKA-31 to mitigate endothelial dysfunction arising from the systemic hypercholesterolemia and metabolic syndrome present in these mice. A recent study by Back and colleagues has reported that SKA-31 treatment prevents platelet aggregation (Back et al., 2022), which may also contribute to the beneficial effects of SKA-31 administration on endothelial function in atherosclerotic mice by reducing thrombogenesis and/or plaque development/progression. However, our study did not evaluate this potential endpoint.

In summary, our results demonstrate that prolonged administration of the K_Ca_ channel activator SKA-31 improved aortic endothelial function in the setting of atherogenesis, but did not decrease atherosclerotic fatty plaque area. These findings set the stage for future studies to elucidate the mechanism behind this effect, its longer-term consequences on aortic stiffness and cardiac performance and to explore whether improvement of aortic endothelial function by K_Ca_ channel activators can promote fatty plaque stability, as is the case with lipid-lowering statin drugs.

## Data Availability

The raw data supporting the conclusions of this article will be made available by the authors, without undue reservation.
