# Supplementary material for: Administration of the KCa channel activator SKA-31 improves endothelial function in the aorta of atherosclerosis-prone mice
Source: Front Pharmacol. 2025 Feb 28;16:1545050. doi: 10.3389/fphar.2025.1545050 (PMC11906683; doi:10.3389/fphar.2025.1545050)
Supplement: Supplementary file 1 [file DataSheet1.pdf]

Supplementary Information for manuscript:

Administration of the K_{Ca} channel activator SKA-31 improves endothelial function in the aorta of atherosclerosis-prone mice

O. Daniel Vera[1], Ramesh C. Mishra[1], Rayan Khaddaj-Mallat[1], Liam Hamm[1], Barak Almarzouq[1], Yong-Xiang Chen[2], Darrell Belke[2], Heike Wulff[3] and Andrew P. Braun[1,2]

[1]Dept. of Physiology and Pharmacology and [2]Libin Cardiovascular Institute, Cumming School of Medicine, University of Calgary, Canada and [3]Dept. of Pharmacology, School of Medicine, University of California Davis USA

Supplementary Table 1: Aortic root to femoral artery pulse wave velocity (PWV) measured via M-mode echocardiography.

Parameters		WT Normal Diet (n=7)	$Apoe^{-/-}$ HFD + Vehicle (n=11)	$Apoe^{-/-}$ HFD + SKA-31 (n=11)	$Apoe^{-/-}$ HFD + Senicapoc (n=11)
PWV	Baseline	ND	3.66±0.24	3.59±0.40	3.57±0.62
(m/s)	Terminal	3.54±0.14	3.65±0.41	3.54±0.27	3.67±0.39

ND: Not Determined. Baseline PWV and terminal PWV values were compared amongst the four groups at the same time points via a one-way ANOVA, followed by a Tukey's post-hoc test. The baseline and terminal PWV values within each $Apoe^{-/-}$ HFD group were compared via a two-tailed paired Student's t-test. Data are presented as mean ± SD.

Supplementary Table 2: Left ventricular cardiac functional and structural parameters measured by echocardiography at the baseline and terminal time points of the treatment protocol.

Parameters		WT Normal Diet (n=7)	$Apoe^{-/-}$ HFD + Vehicle (n=11)	$Apoe^{-/-}$ HFD + SKA-31 (n=11)	$Apoe^{-/-}$ HFD + Senicapoc (n=11)
EF (%)	Baseline	ND	55.47±7.94	56.95±7.21	53.39±7.46
	Terminal	50.88±4.95	54.07±8.94	56.68±8.61	54.63±11.41
FS (%)	Baseline	ND	28.75±5.60	29.61±4.82	27.34±4.88
	Terminal	25.91±3.05	27.84±5.72	29.53±5.82	28.33±7.48
CO (mL/min)	Baseline	ND	11.05±2.78	9.60±2.20	9.75±2.35
	Terminal	12.37±2.49	10.75±2.72	10.00±2.87	9.63±1.68
SV (μL)	Baseline	ND	30.22±6.02	26.98±4.21	30.06±6.89
	Terminal	35.43±4.50	28.69±5.35*	27.91±5.12	26.79±2.33
EDV (μL)	Baseline	ND	58.39±8.83	53.78±6.24	56.32±11.87
	Terminal	73.98±4.39	55.52±7.24***	55.82±6.69	56.79±11.91
ESV (μL)	Baseline	ND	28.17±7.31	26.80±6.26	29.29±7.03
	Terminal	38.56±5.37	26.83±8.81*	27.91±8.47	30.00±11.00
HR (bpm)	Baseline	ND	372.10±59.30	361.20±43.27	343.50±31.89
	Terminal	356.20±29.06	376.20±42.00	366.50±54.17	354.40±50.63
LVPW;d (mm)	Baseline	ND	0.83±0.10	0.79±0.06	0.86±0.16
	Terminal	0.86±0.15	0.86±0.10	0.85±0.15	0.82±0.11

ND: Not Determined; EF: Ejection Fraction; FS: Fractional Shortening; CO: Cardiac Output; SV: Stroke Volume; EDV: End Diastolic Volume; ESV: End Systolic Volume; HR: Heart Rate in beats per minute (bpm); LVPW;d: Left Ventricular Posterior Wall Thickness measured at Diastole. The four experimental groups were compared at the baseline and terminal time points

via a one-way ANOVA, followed by a Tukey's post-hoc test (i.e., across a row). The baseline and terminal parameters within each Apoe$^{-/-}$ HFD group were compared via a two-tailed paired Student's t-test (i.e., within a column). Data are presented as mean ± SD. *$P<0.05$, ***$P<0.001$ vs. WT Normal Diet Terminal. Among the three Apoe$^{-/-}$ HFD treatment groups, no statistical differences were noted for a listed parameter, or between the baseline and terminal values within a given Apoe$^{-/-}$ HFD group.

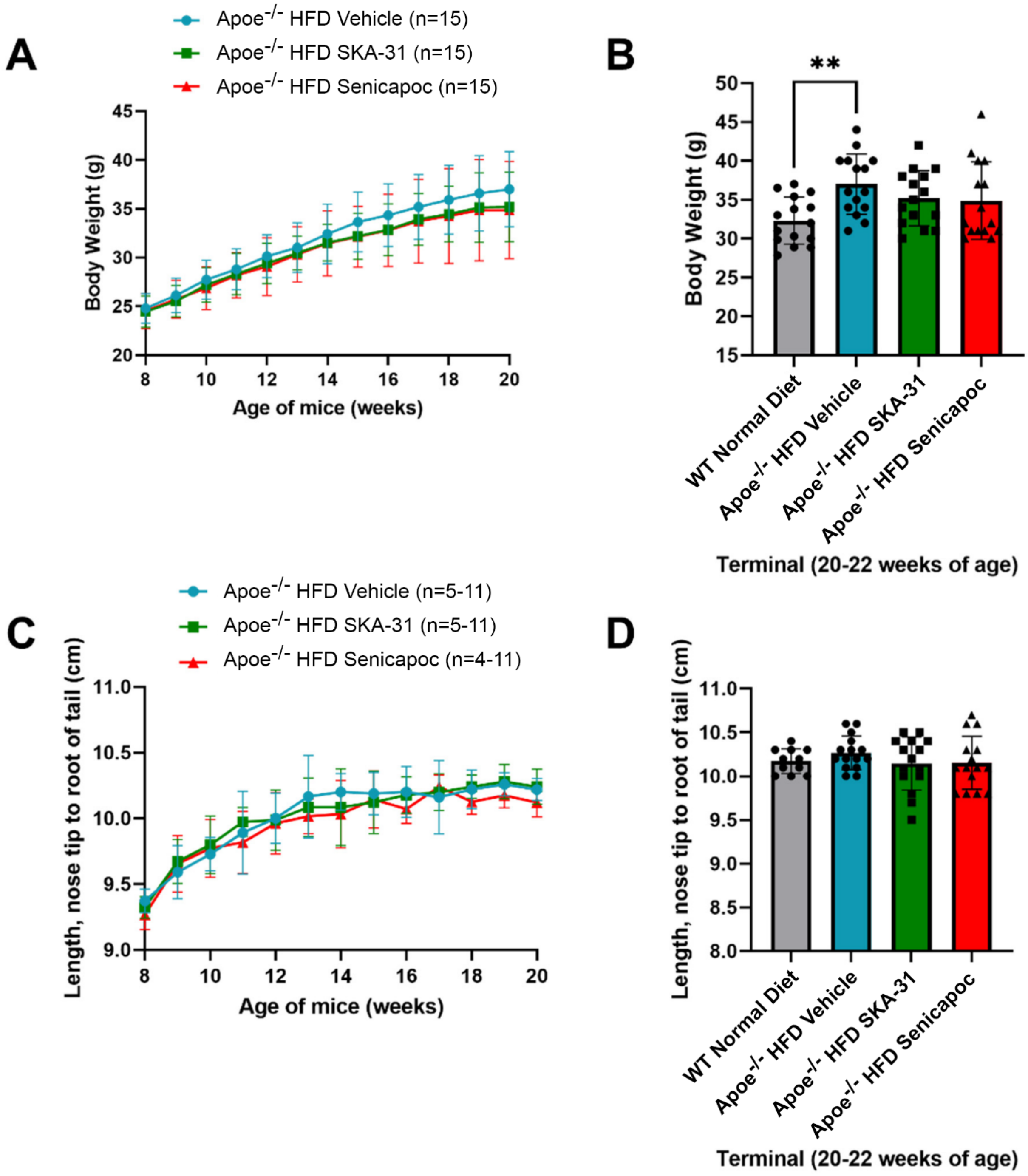

Supplementary Figure 1 - Quantification of total body weight and body length in Apoe$^{-/-}$ HFD mice over the time course of vehicle and drug administration.

(A) Time course of body weight gain in Apoe$^{-/-}$ HFD Vehicle, Apoe$^{-/-}$ HFD SKA-31, and Apoe$^{-/-}$ HFD senicapoc mice from 8 weeks of age (baseline) until ~20 weeks of age (terminal,

total of 12 weeks of vehicle/drug treatment). **(B)** Body weight of Apoe$^{-/-}$ HFD mice and WT Normal Diet mice at the terminal point (20-22 weeks of age). **(C)** Time course of growth-related increase in body length (nose tip to root of tail) in Apoe$^{-/-}$ HFD Vehicle, Apoe$^{-/-}$ HFD SKA-31, and Apoe$^{-/-}$ HFD senicapoc mice from 8 weeks of age (baseline) until 20 weeks of age. Note that the average data plotted at the 8-week time point were obtained from 4-5 mice per group; measurements at all subsequent time points reflect 11 mice per group. **(D)** Body length, nose tip to root of tail, of Apoe$^{-/-}$ HFD mice and WT Normal Diet mice at the terminal point (20-22 weeks of age). Data in panel A were analyzed with a two-way, repeated measures ANOVA and Tukey's post-hoc test. Data in panel C were subjected to a mixed-effects analysis and a Tukey's post-hoc test. Data in panels B and D were analyzed with a one-way ANOVA, followed by a Tukey's post-hoc test. All results are presented as mean ± SD, with n=4-15 mice per group, $^{**}P<0.01$.

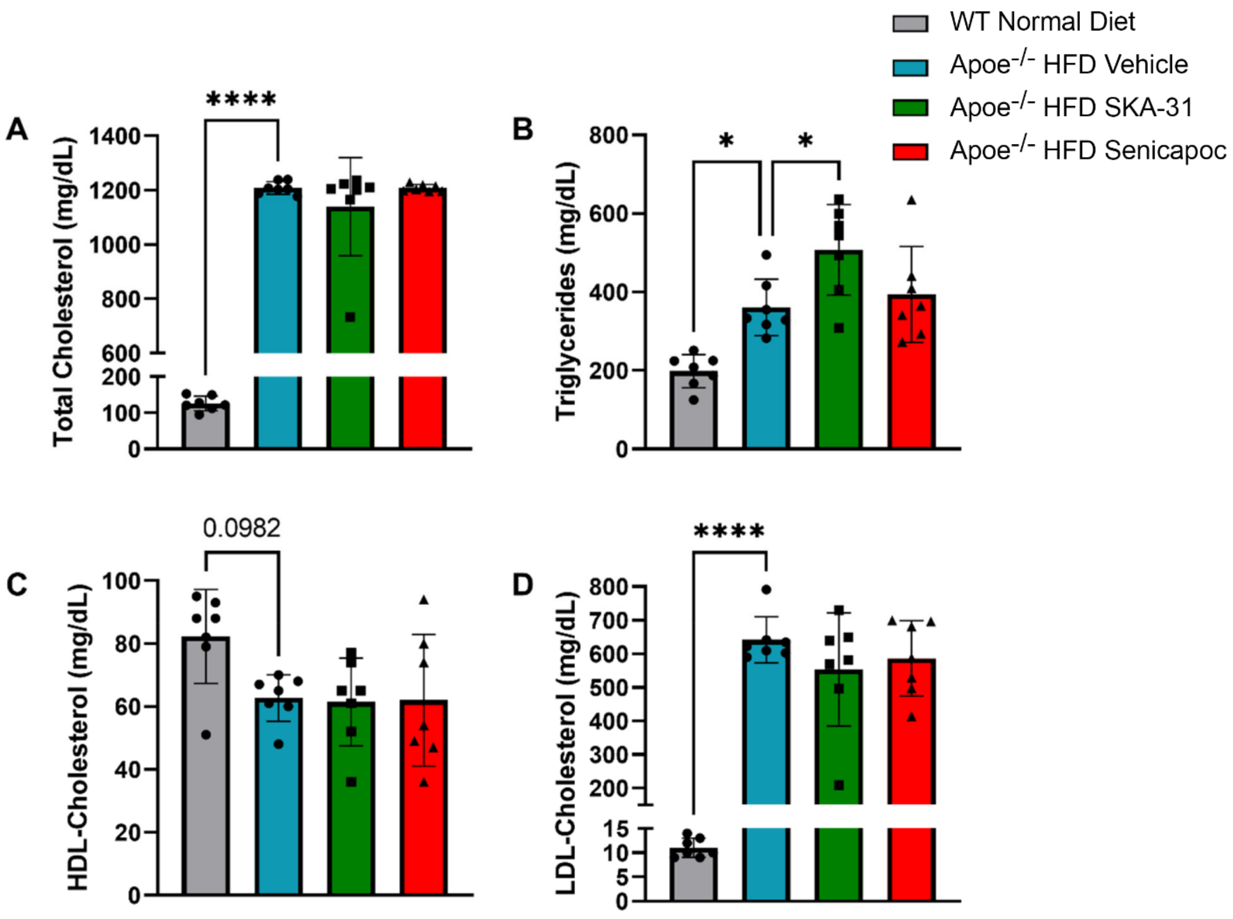

Supplementary Figure 2 - Total cholesterol, triglycerides, HDL-cholesterol, and LDL-cholesterol levels in the blood serum of WT mice and Apoe$^{-/-}$ HFD mice.

(A) Total cholesterol, **(B)** triglycerides, **(C)** HDL-cholesterol, and **(D)** LDL-cholesterol concentrations in blood serum taken from WT and Apoe$^{-/-}$ HFD mice after the terminal point of the protocol. Data points represent measurements from individual animals in each group; analysis of individual serum components was performed by IDEXX Bio-Analytics (Sacramento, CA, USA) using a colorimetric assay. Data are presented as mean ± SD and were analyzed with a one-way ANOVA, followed by a Tukey's post-hoc test. N=7 mice per group, **P*<0.05; *****P*<0.0001.

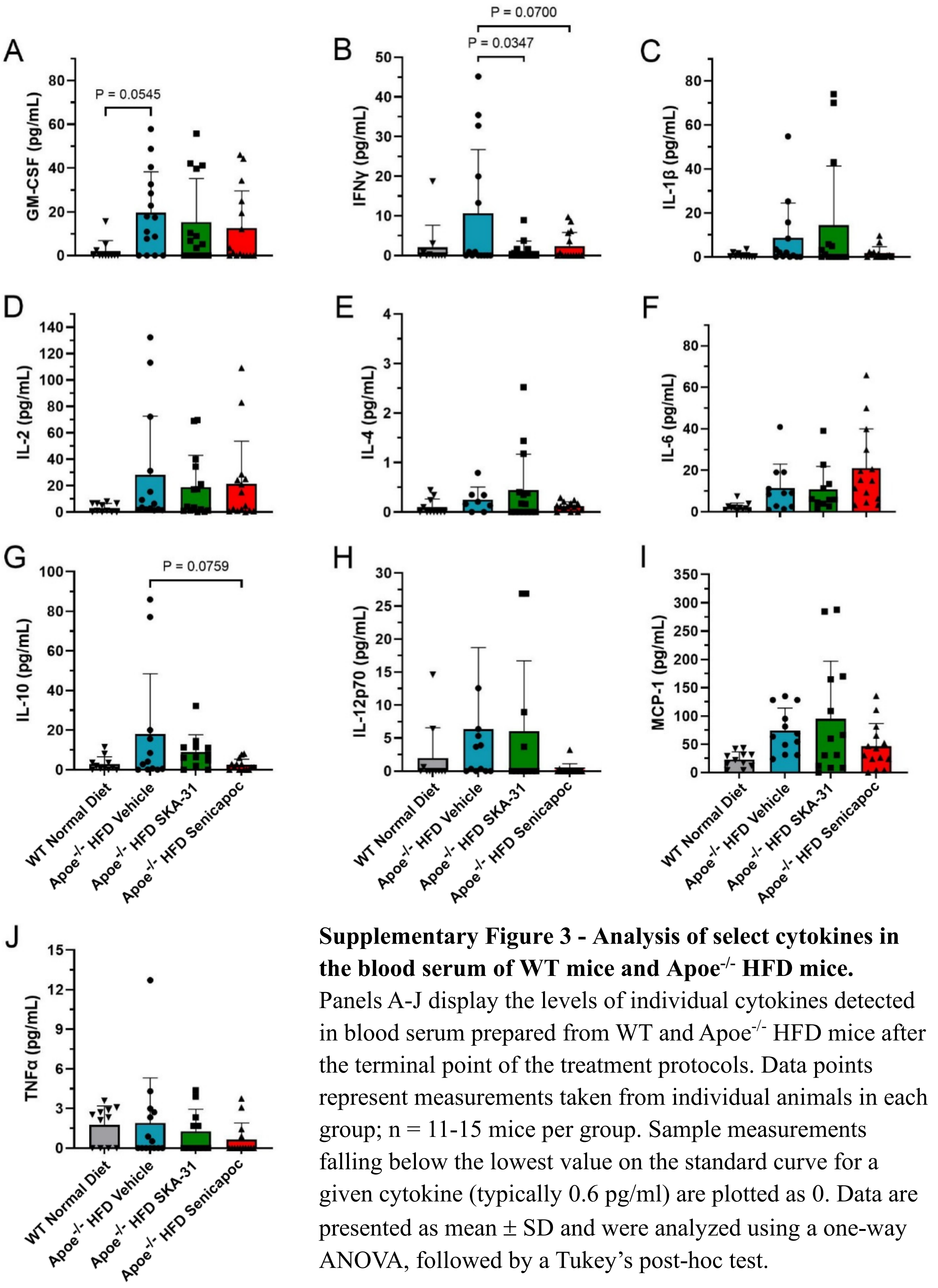

Supplementary Figure 3 - Analysis of select cytokines in the blood serum of WT mice and Apoe$^{-/-}$ HFD mice. Panels A-J display the levels of individual cytokines detected in blood serum prepared from WT and Apoe$^{-/-}$ HFD mice after the terminal point of the treatment protocols. Data points represent measurements taken from individual animals in each group; n = 11-15 mice per group. Sample measurements falling below the lowest value on the standard curve for a given cytokine (typically 0.6 pg/ml) are plotted as 0. Data are presented as mean ± SD and were analyzed using a one-way ANOVA, followed by a Tukey's post-hoc test.

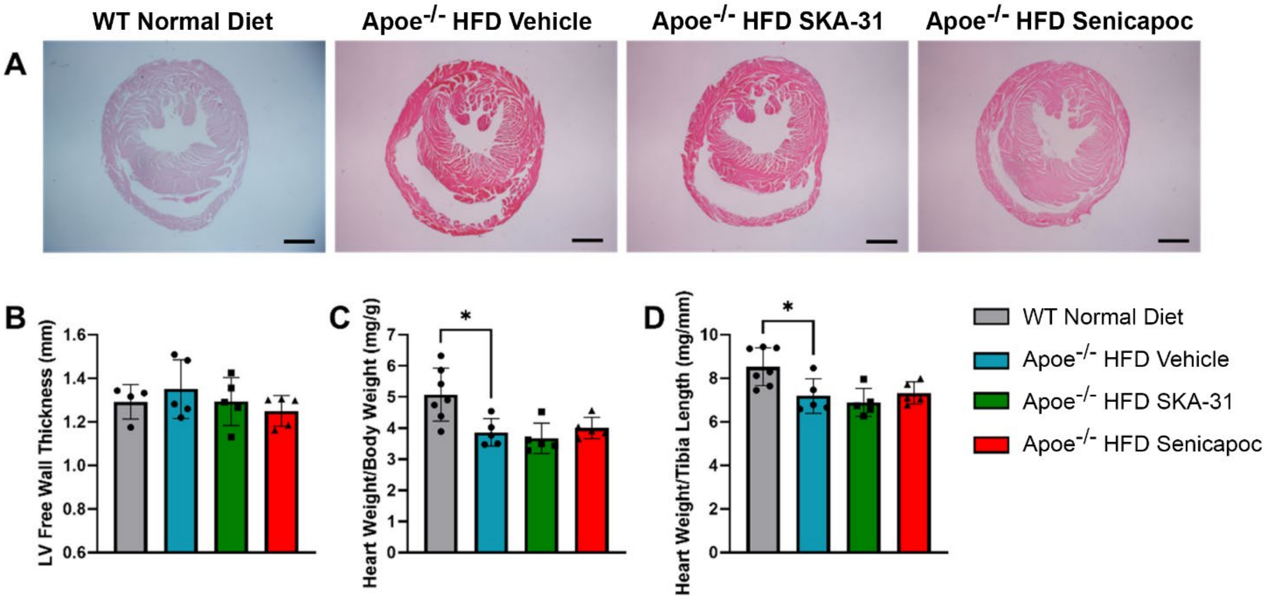

Supplementary Figure 4 – Left ventricular free wall thickness and cardiac size measurements in WT and Apoe$^{-/-}$ HFD mice.

(A) Representative H&E-stained mid-myocardium cross-sections from WT Normal Diet mice, Apoe$^{-/-}$ HFD Vehicle-treated mice, Apoe$^{-/-}$ HFD SKA-31-treated mice and Apoe$^{-/-}$ HFD senicapoc-treated mice. Scale bar: 1 mm. **(B)** Left ventricular (LV) free wall thickness measured in the H&E-stained mid-myocardium cross-sections. **(C)** Heart Weight/Body Weight ratio, presented as mg/g. **(D)** Heart Weight/Tibia Length ratio, presented as mg/mm. Data in panels B-D are presented as mean ± SD, with a N value of 4-7 mice for the WT Normal Diet group, and N=5 mice for each of the Vehicle, SKA-31 and senicapoc treatment groups. Data were analyzed using a one-way ANOVA, followed by a Tukey's post-hoc test; *P<0.05.

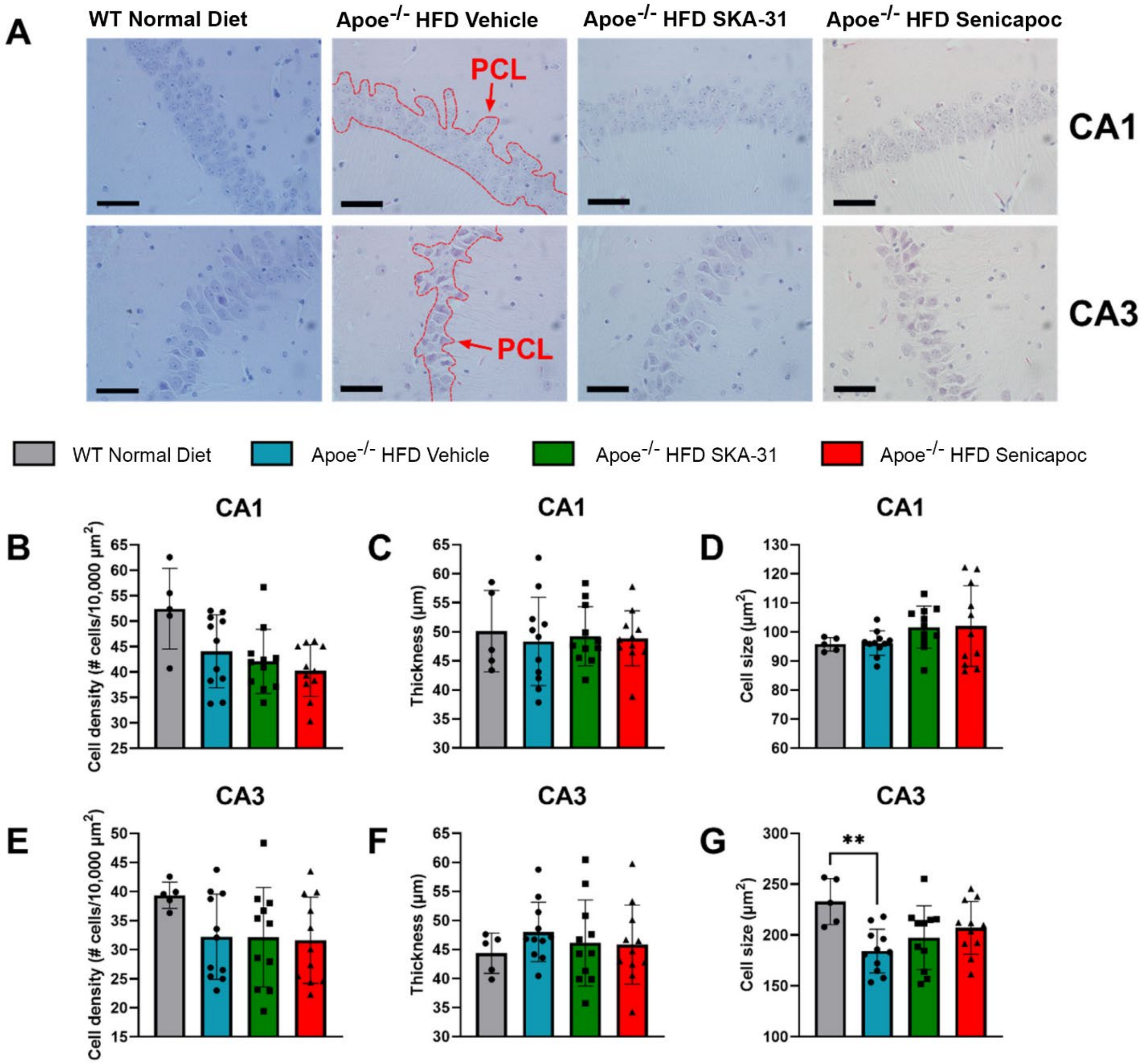

Supplementary Figure 5 - Measurements of hippocampus CA1 and CA3 pyramidal cell density, pyramidal cell layer thickness, and pyramidal cell size in WT mice and Apoe$^{-/-}$ HFD mice.

(**A**) Representative H&E-stained sections of the CA1 and CA3 hippocampus regions of WT Normal Diet mice (n=5), Apoe$^{-/-}$ HFD Vehicle-treated mice (n=11), Apoe$^{-/-}$ HFD SKA-31-treated mice (n=11), and Apoe$^{-/-}$ HFD senicapoc-treated mice (n=11). The top row shows the CA1 region, and the bottom row shows the CA3 region. The pyramidal cell layer (PCL) is outlined in red in the representative images for Apoe$^{-/-}$ HFD Vehicle to assist the reader with visualization of

the PCL. Scale bar: 50 μm. Cell density **(B)**, thickness **(C)**, and cell size **(D)** for the CA1 PCL, as well as cell density **(E)**, thickness **(F)**, and cell size **(G)** for the CA3 PCL, are shown. Data in panels B-G are presented as mean ± SD and were analyzed with a one-way ANOVA, followed by a Tukey's post-hoc test; $^{**}P<0.01$.

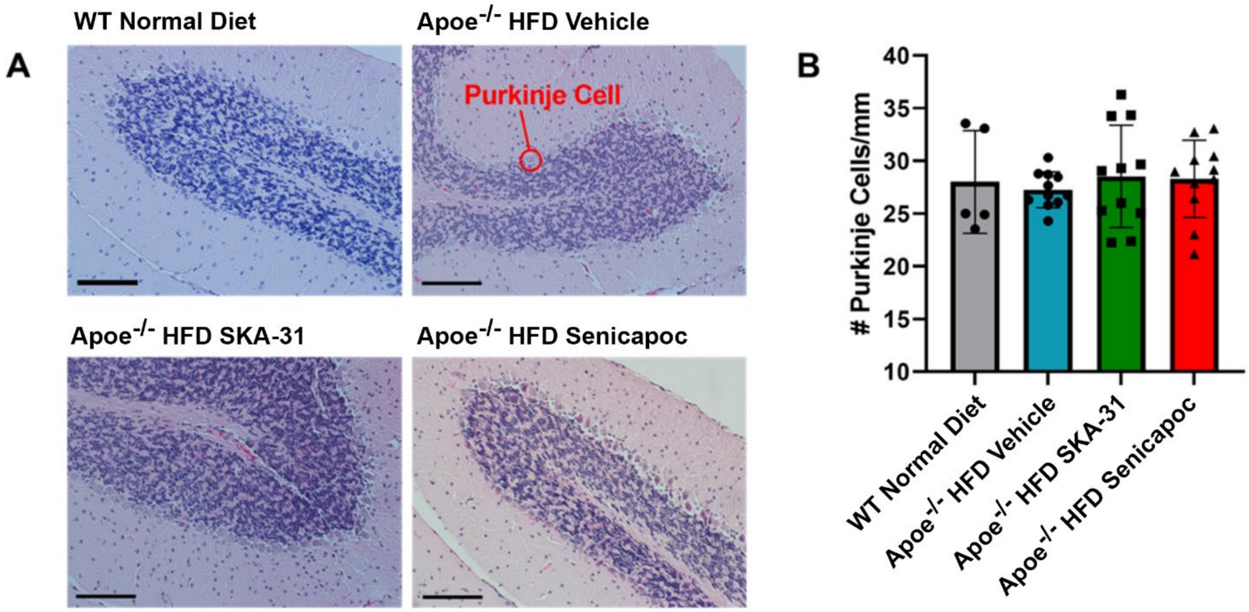

Supplementary Figure 6 - Measurement of Purkinje cell density in the cerebellum of WT mice and Apoe$^{-/-}$ HFD mice.

(A) Representative H&E-stained sections of the cerebellum from WT Normal Diet mice (n=5), Apoe$^{-/-}$ HFD Vehicle-treated mice (n=11), Apoe$^{-/-}$ HFD SKA-31-treated mice (n=11), and Apoe$^{-/-}$ HFD senicapoc-treated mice (n=11). A single Purkinje cell is circled in red in the representative image from the Apoe$^{-/-}$ HFD vehicle treatment group. Scale bar: 100 μm. **(B)** Purkinje cell density, plotted as the number of Purkinje cells/mm length of Purkinje cell layer. Data in B are presented as mean ± SD and were analyzed with a one-way ANOVA, followed by a Tukey's post-hoc test.

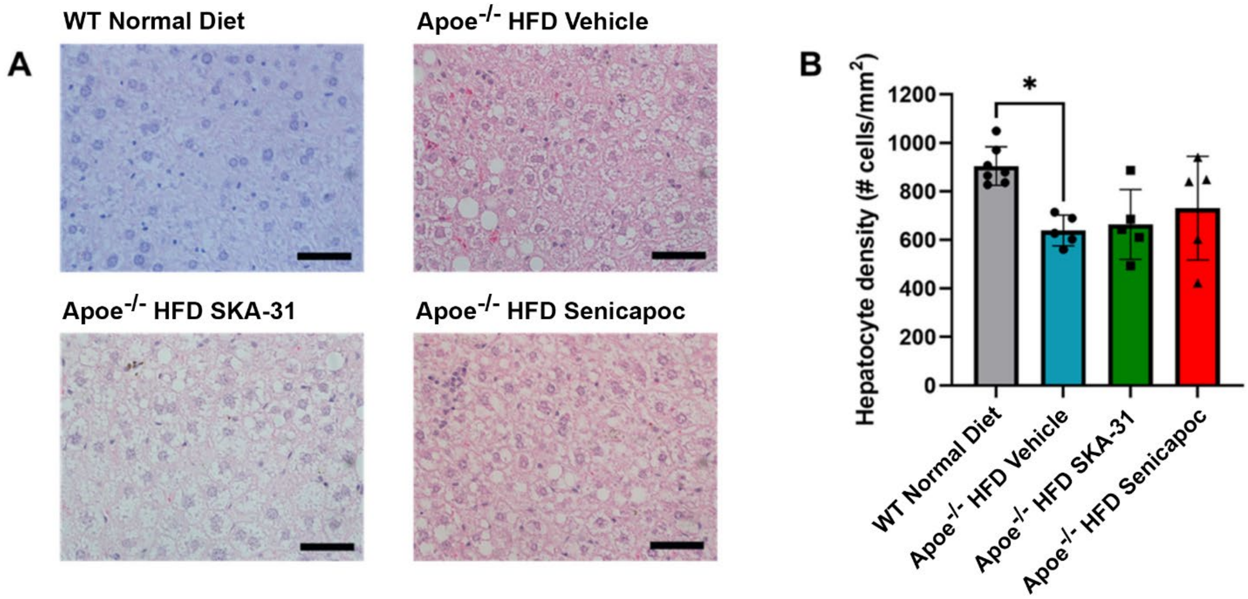

Supplementary Figure 7 - Hepatocyte cell density measurement in the liver of WT and Apoe$^{-/-}$ HFD mice.

(A) Representative H&E-stained liver sections of WT Normal Diet mice (n=7), Apoe$^{-/-}$ HFD Vehicle-treated mice (n=5), Apoe$^{-/-}$ HFD SKA-31-treated mice (n=5), and Apoe$^{-/-}$ HFD senicapoc-treated mice (n=5). Scale bar: 50 µm. **(B)** Hepatocyte density, quantified as the number of cells/mm^2, is plotted for the four groups of mice. Data are presented as mean ± SD and were analyzed with a one-way ANOVA, followed by a Tukey's post-hoc test; *P<0.05.

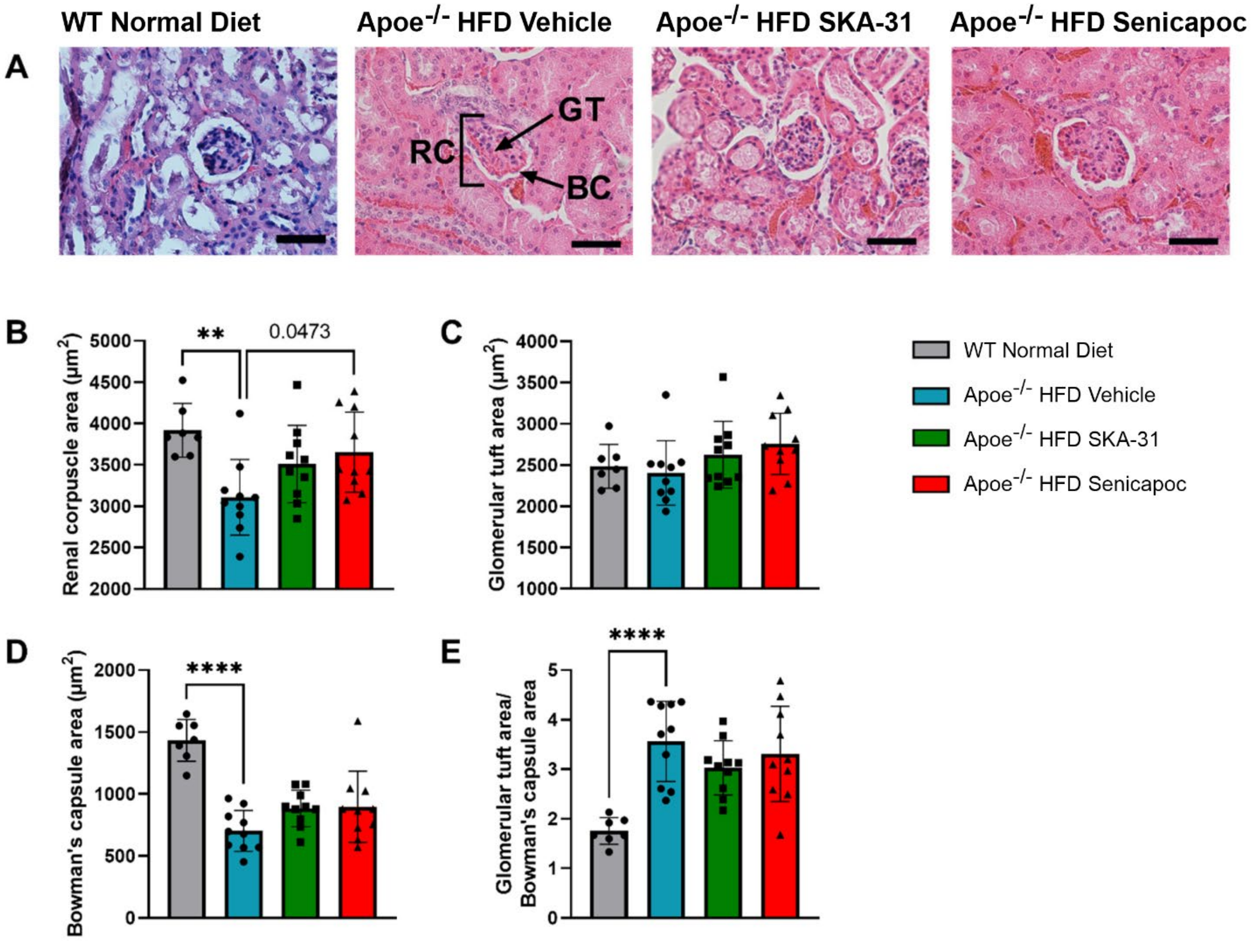

Supplementary Figure 8 - Morphological analysis of the renal glomerulus in kidneys of WT mice and Apoe$^{-/-}$ HFD mice.

(A) Representative H&E-stained sections of renal glomeruli from WT Normal Diet mice (n=7), Apoe$^{-/-}$ HFD Vehicle-treated mice (n=10), Apoe$^{-/-}$ HFD SKA-31-treated mice (n=10), and Apoe$^{-/-}$ HFD senicapoc-treated mice (n=10). The measured parameters of the glomerulus are labelled in the representative image for the Apoe$^{-/-}$ HFD vehicle treatment group; RC: renal corpuscle, GT: glomerular tuft, and BC: Bowman's capsule. Scale bar: 50 µm. **(B)** Renal corpuscle area, consisting of the glomerular tuft area and the Bowman's capsule area added together. **(C)** Glomerular tuft area. **(D)** Bowman's capsule area. **(E)** Ratio of the glomerular tuft area and the Bowman's capsule area. Data are presented as mean ± SD and were analyzed with a one-way ANOVA, followed by a Tukey's post-hoc test; ***P*<0.01, *****P*<0.0001.
